# Kinetics, thermodynamics, and mechanisms of PMO interactions from computational molecular modeling

**DOI:** 10.1016/j.omtn.2026.103056

**Published:** 2026-08-12

**Authors:** Ying Chou, Igor Novikov, Daniel Pierson, Kenneth A. Marx, Arani Chanda, Valeri Barsegov

**Affiliations:** 1 Department of Chemistry, University of Massachusetts, Lowell, MA 01854, USA; 2 Sarepta Therapeutics, Cambridge, MA 02142, USA

**Keywords:** MT: oligonucleotides: therapies and applications, phosphorodiamidate morpholino oligomers, biomolecular interactions, viscosity, molecular dynamics simulations, computational molecular modeling, antisense oligonucleotides, formulation, Duchenne muscular dystrophy

## Abstract

Phosphorodiamidate morpholino oligonucleotides (PMOs) are a class of antisense oligonucleotides. While PMOs have enabled several nucleic acid therapeutics, their structural and energetic solution properties remain poorly understood. Using solution viscosity measurements combined with computational molecular modeling, we explored interactions of therapeutic 22-mer, 25-mer, and 30-mer PMOs in concentrated solutions. Self-association of PMO monomers involves non-specific interactions, is energetically favorable (with estimates of −32 to −67 kcal/mol interaction energies), and exhibits biphasic kinetics involving a fast phase of hydrophobic anchoring followed by a slower phase associated with optimization of intermolecular base pairing and stacking interactions. The final complexes possess broad self-association interfaces of ∼700–1,600 Å^2^. Accurate interpretation of the viscosity vs. concentration data for concentrated PMO solutions must account for PMO dimer formation, as supported by the molecular dynamics simulations. Formation of higher-order PMO species was inferred from viscosity-concentration profiles based on models 1–4: for the 25-mer at concentrations above 160 mg/mL (dimerization) and 240 mg/mL (trimerization), and for the 30-mer above 190 mg/mL (dimerization) and 270 mg/mL (trimerization), respectively. The results provide atomic-level details on PMO structure, molecular properties, and interaction energies in concentrated environments, identifying weak preferential functional group interaction patterns that underlie thermodynamic stability.

## Graphical abstract

**Figure:**
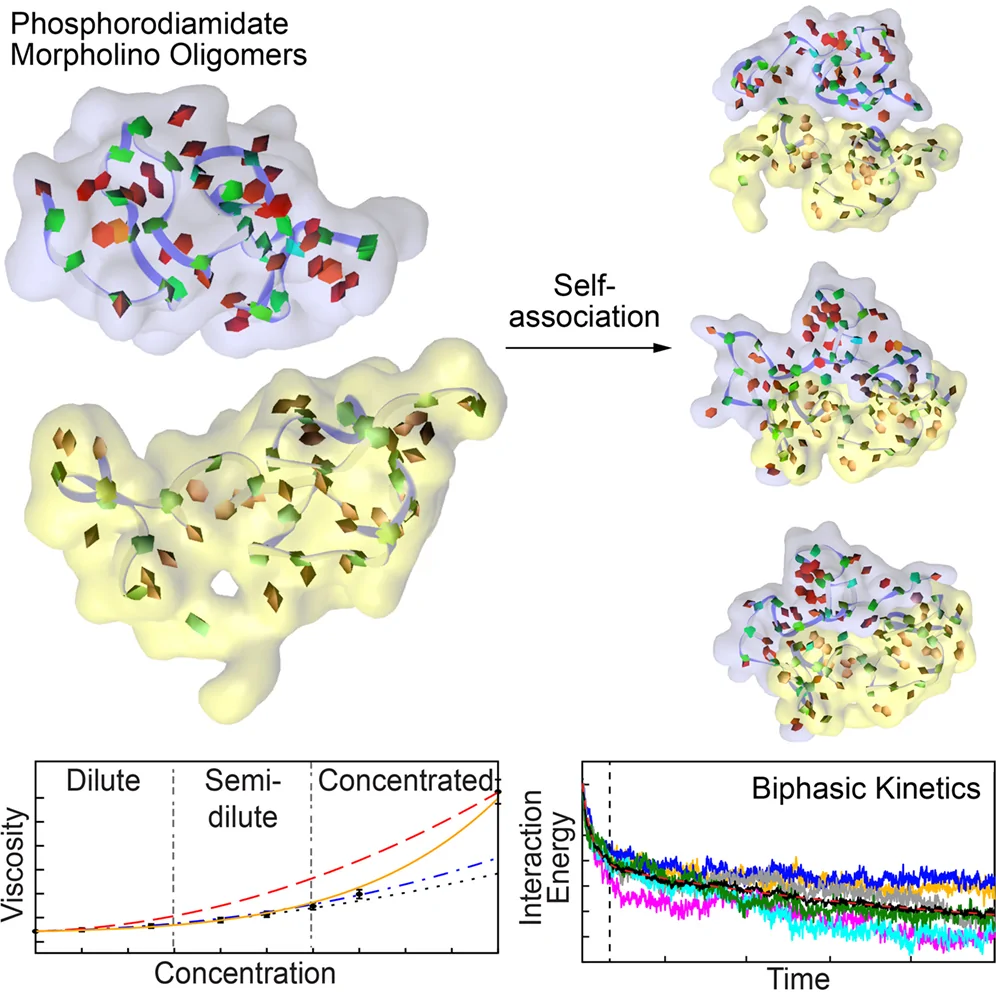

## Teaser

Phosphorodiamidate morpholino oligonucleotide (PMO) therapeutics exhibit favorable self-association in concentrated solutions through hydrophobic, base-pairing, and stacking interactions. Combining viscosity measurements with molecular dynamics simulations, Chou and colleagues reveal biphasic PMO association kinetics, broad intermolecular interfaces, and molecular interaction patterns that inform formulation strategies to minimize aggregation and preserve solution stability.

## Introduction

Single-stranded nucleic acids have long been studied, initially to explore the structural variability of natural DNA and RNA, and later to mimic the functions of specific single-stranded RNAs to understand their roles in normal cellular functions, disease etiology, and progression.^1^^,^^2^^,^^3^^,^^4^ Early efforts to use canonical RNA therapeutically for gene editing,^5^ replacement, addition, or inhibition were limited by issues such as immune activation,^5^ rapid nuclease degradation,^6^ and delivery challenges.^7^ These obstacles prompted the development of modified backbones,^8^^,^^9^ leading to diverse nucleic acid analog platforms and several FDA-approved therapeutics.^10^^,^^11^

**Figure 1:**
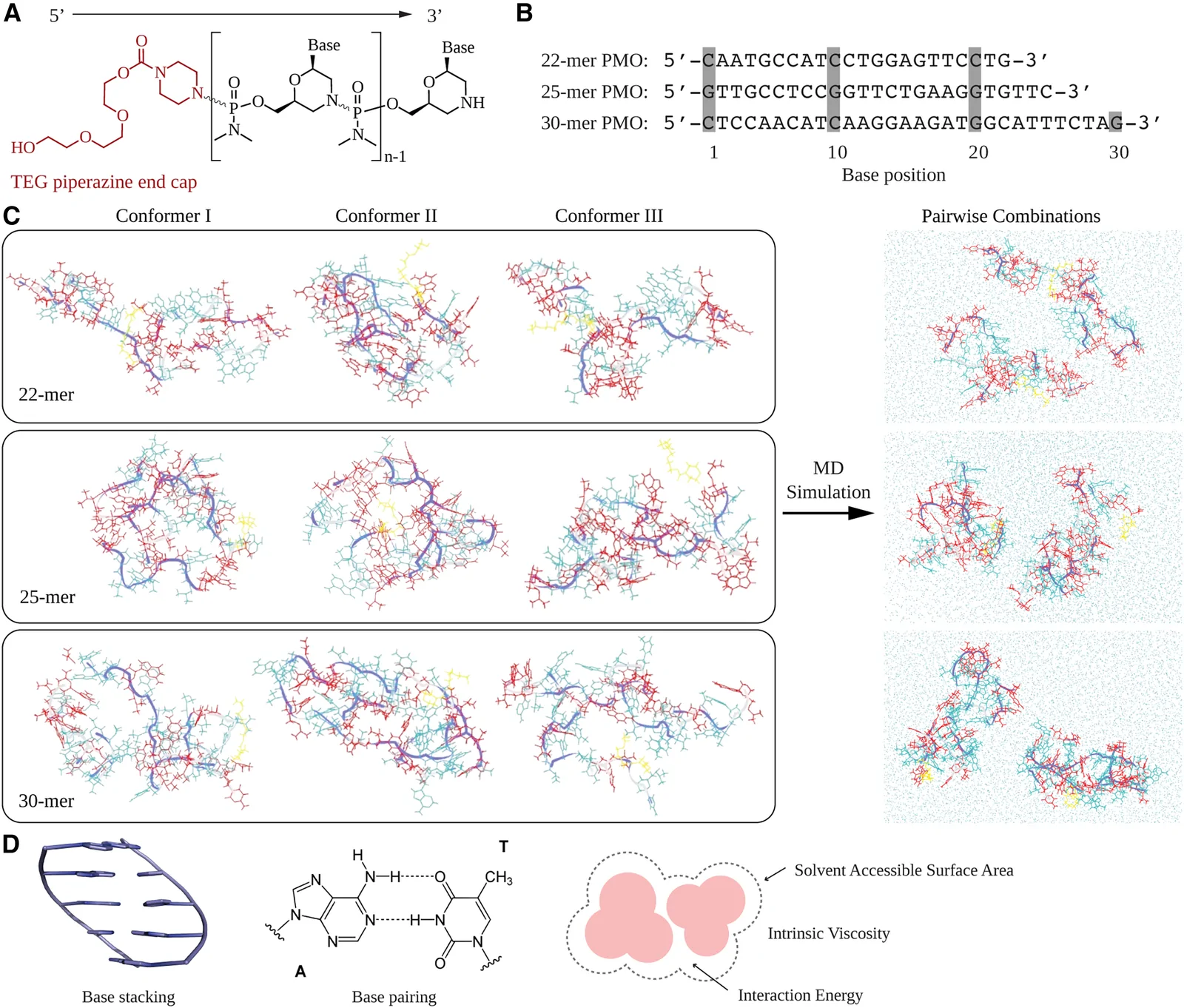
Structures of therapeutic 22-mer, 25-mer, and 30-mer PMOs and construction of PMO dimers (A) Representative structures and sequences of the three therapeutic PMOs: for Exon45 (22-mer PMO), Exon53 (25-mer PMO), and Exon51 (30-mer PMO), each featuring a 5′-end conjugated to a morpholino triethylene glycol (TEG) piperazine end cap. (B) The sequences of ‘morpholino nucleobases’ for 22-mer, 25-mer, and 30-mer PMOs. (C) Homodimeric and heterodimeric PMO conformer structures reconstituted by pairing the most representative conformers I, II, and III for each PMO length. Bases are shown as lines (red: A, T; cyan: C, G); TEG piperazine end cap as yellow lines; backbones as twisted ribbons. Also shown in solvated boxes (on the right) with water (blue dots) are examples of the initial structures used in the all-atom MD simulations for three pairwise combinations of conformers I and II for 22-mer PMO, conformers I and III for 25-mer PMO, and conformers II and III for 30-mer PMO. (D) Examples of data analyses based on the output from the all-atom MD simulations carried out for each PMO dimer system in aqueous solution at room temperature.

Phosphorodiamidate morpholino oligonucleotides (PMOs) (Figure 1) are a class of antisense oligonucleotides (ASOs) in which the standard nucleic acid backbone is replaced with morpholino rings linked by phosphorodiamidate bonds.^12^^,^^13^^,^^14^ PMOs exhibit high solubility, resistance to metabolic degradation, and strong duplex stability, making them well-suited for therapeutic use.^13^^,^^15^ Since 2016, PMOs have been FDA-approved for Duchenne muscular dystrophy (DMD)^16^^,^^17^^,^^18^ and have also been designed to target Marburg, Ebola,^19^^,^^20^ other viruses,^15^^,^^21^^,^^22^ and bacterial pathogens.^23^ In DMD therapy, PMOs bind to dystrophin pre-mRNA to induce exon skipping, restoring the reading frame and enabling production of a shortened yet functional dystrophin protein. Through Watson-Crick base pairing, PMOs bind target mRNA and block translation via an RNase H-independent steric mechanism.^23^ Similarly, PMOs can inhibit viral replication by preventing translation of viral RNA genomes.^15^ They are resistant to degradation by nucleases, proteases, esterases, and hydrolases,^24^ and their uncharged backbones minimize protein binding, reducing off-target effects and improving safety.^23^^,^^25^

Although PMOs possess these attractive properties, which make them stand out from DNA, RNA, and other ASOs in terms of their solution structures, roles in therapeutic activities and interactions with other biological moieties, very little is known about PMOs’ dynamic structural properties. Until recently, there was no information about the specific PMO structures that exist in aqueous solution. To date, there are no experimentally resolved X-ray crystals or NMR structures that exist for PMO oligomers. Only one study on a related morpholino backbone has been published on the oligomers’ physical properties^26^; yet, no structural information was revealed. Other methods available to researchers, such as small-angle X-ray scattering (SAXS), analytical ultracentrifugation (AUC), and dynamic light scattering (DLS), do not provide structural information anywhere near the atomic level of details. SAXS measurements carry information about the distribution of interatomic distances (not atomic structure), which can be used to probe the molecular size and shape, while AUC and DLS provide size and gross shape information.

In our pioneering study of therapeutics-based PMOs comprised of 22 nucleobases (22-mer), 25 nucleobases (25-mer), and 30 nucleobases (30-mer),^27^ we combined their experimental circular dichroism (CD) and solution viscosity measurements with the all-atom molecular dynamics (MD) simulations of them in solution, followed by machine learning to resolve, for the first time, solution structures of the 22-mer, 25-mer, and 30-mer length PMO molecules. These three oligomers’ sequences are complementary to Exon 45, Exon 53, and Exon 51, respectively, of the dystrophin gene pre-mRNA transcript.^28^ First, we calculated atomic partial charges and developed the atomic force field for those atoms and atomic groups forming the morpholino ring and the phosphorodiamidate group in the PMO backbone. These efforts enabled us to then carry out microsecond-long MD simulations of the 22-mer, 25-mer, and 30-mer PMOs. Using atomic resolution inherent in the all-atom MD approaches, we found that, owing to the charge-neutral nature of PMOs, their structures are very different from DNA, RNA, and other ASOs. Interestingly, these PMOs formed an ensemble of solution structures rather than a single structure, and their conformational dynamics are mainly defined by the competition between nonpolar nucleobases and uncharged phosphorodiamidate groups for shielding from solvent exposure. As a result, PMO molecules form non-canonical, well-hydrated (good solubility), partially helical, yet thermodynamically stable folded structures.^27^

Building on the success achieved in this first study of therapeutic PMO molecules,^27^ we recently used a similar approach to explore the structure-function relationships for the therapeutic peptide (-GlyArg_6_) conjugated phosphorodiamidate morpholino oligonucleotides (PPMOs).^29^ We found that the secondary structure arrangement of the PMO components of PPMOs is not affected by the presence of conjugated -GlyArg_6_ peptide. Similar to the case of PMO molecules, the non-canonical partially helical, folded structures of the PPMO molecules are characterized by a small radius of gyration and a low count of both base pairs and base stacks. We also mapped the position-dependent intramolecular interactions between the PMO and peptide components of PPMOs, which do not affect the PPMOs’ ability to hybridize, but decrease the viscosity of PPMOs’ aqueous solutions as compared with PMOs’ solutions.^29^ More recently, using the all-atom MD-based molecular modeling, we probed the interaction of PMO molecules with mild surfactants Polysorbate 80 and Polysorbate 20.^30^ We found that while the PMO-surfactant (non-covalent) interactions do not significantly perturb the PMO conformers’ secondary structure base-pairing and base-stacking arrangements, and their chirality, in full agreement with experimental CD spectra, they enlarge the overall PMO conformers’ tertiary structure. Interestingly, the Polysorbate 80 and Polysorbate 20 molecules, especially their most hydrophobic arms, exhibit limited preferential interactions at specific positions in these three PMOs.

**Figure 2:**
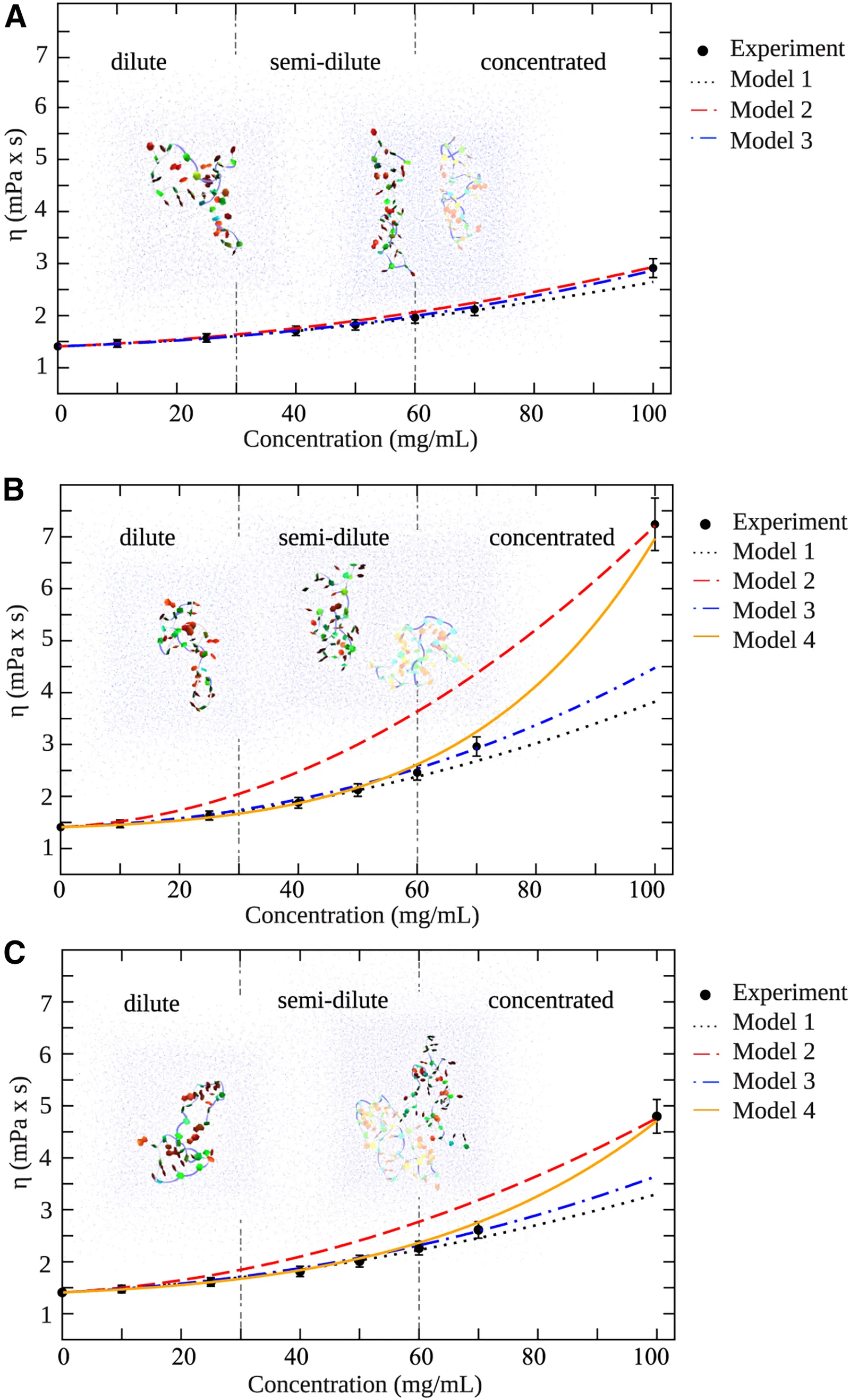
Theoretical reconstruction of viscosity profiles for 22-mer, 25-mer, and 30-mer PMOs Superposed are the 25°C viscosity *η* versus concentration *C* profiles for 22-mer (A), 25-mer (B), and 30-mer (C) PMO solutions obtained experimentally (black data points with standard deviations) and theoretically (curves). The dotted black curve and dashed red curve are the results obtained using the quadratic version of the Huggins Equation 1 for the monomeric PMO (model 1) and dimeric PMO (model 2), respectively. The dash-dotted blue curve and solid orange curve are theoretical predictions obtained with quadratic version (Equation 1) and cubic version (Equation 2) of the Huggins equation together with the models which account for PMO dimerization (model 3) and PMO trimerization (model 4), respectively.

To broaden our understanding of the structure-function relationship for therapeutic PMOs, in this study, we explored the molecular signatures and energetics of PMO-PMO interactions in aqueous solutions (Figure 1), which underly the non-linear dependence of PMO experimental solution viscosity on solution concentration in the concentrated solution regime (>75 mg/mL; Figure 2). We further mapped and analyzed PMO-PMO interactions over time to understand the biphasic kinetics and two-step mechanism involved in their complex formation. While experimental approaches, in general, are limited in their ability to resolve the molecular mechanisms underlying PMO-PMO interactions, particularly at high concentrations, simple viscosity characterization continues to be an important physical property of PMO oligomers to understand PMO solution aggregation propensity. These properties are directly relevant to the stable and injectable formulation aspects of PMO therapeutics.^31^^,^^32^ Maintaining structural integrity and controlling solution viscosity are essential for PMO therapeutic efficacy, as elevated viscosity can increase the risk of aggregation, making formulation stability a key concern in pharmaceutical development. Viscosity measurements provide information about the size, shape, and rigidity of biomolecular complexes,^33^ which can be correlated with solution structures of PMO-PMO complexes readily accessible in MD simulations.

The novel structural insights gained on the PMO-PMO interactions in concentrated aqueous solution, combined with detailed atomic-level information about the molecular properties and thermodynamic characteristics underlying formation of the PMO-PMO homo- and heterodimers from similar or different 22-mer, 25-mer, and 30-mer PMO conformers, significantly advance our understanding of the pharmaceutically relevant quality attributes of PMO molecules. The results obtained can be useful in the rational design of a new generation of RNA-mimic drugs. The PMO-PMO interaction energies resolved in this study provide a measure of thermodynamic stability of PMO complexes. The MM/GBSA-based calculations provide first theoretical estimates of interaction energy values, and so these values provide an important energetics reference frame for physico-chemical characterization of PMO molecules. These insights help inform the choice of surfactants or other excipients in mitigating PMO aggregation and changes in PMO solution properties during formulation development.

## Results

### Morpholino “nucleotide” sequences of 22-mer, 25-mer, and 30-mer PMOs

The sequences and structures of three therapeutic PMOs, consisting of 22, 25, and 30 nucleobases, are displayed in Figures 1A and 1B. Each PMO includes a morpholino backbone with standard nucleobases (adenine, cytosine, guanine, and thymine) and a 5′-end morpholino triethylene glycol (TEG) piperazine modification (Figure 1A). The morpholino nucleotide sequences, complementary to Dystrophin pre-mRNA Exons 45 (22-mer), 53 (25-mer), and 51 (30-mer), are shown in Figure 1B. None of the PMOs contains more than two consecutive guanines, making the formation of guanine-rich secondary structures^34^ unlikely, which is consistent with our previous studies, which showed that these PMOs exhibit weak propensity to form canonical secondary structures, exhibiting a low number of base pairs (3–6) and base stacks (6–9).^27^

### Viscosity-concentration profiles

These viscosity-concentration profiles are displayed in Figure 2. While in the dilute and semi-dilute solution regimes (*C*< 60 mg/mL), *η* depends on *C* almost linearly, the dependence of *η* on *C* is non-linear in the concentrated solution (*C*> 60 mg/mL) for all three PMOs (Figure 2). This can be understood by analyzing the expression for specific viscosity $\eta_{sp}=\frac{\eta -\eta_{0}}{\eta_{0}}$ obtained from the perturbative expansion (see Equation 1). When the expansion for *η* is truncated at the quadratic term in *C*, the expression for *η*_*sp*_ becomes: *η*_*sp*_ = $\left[\eta \right]C+{k_{H}\left[\eta \right]}^{2}C^{2}$. In dilute solution (*C*< 20 mg/mL), the quadratic *C*^2^-term is small compared to the linear *C*-term, and the dependence of *η*_*sp*_ on *C* is linear. In concentrated solution (*C*> 60 mg/mL), the *C*^2^-term becomes large, and the dependence of *η*_*sp*_ on *C* is non-linear. In semi-dilute solution range (30 mg/mL <*C*< 60 mg/mL), the *C*-term and *C*^2^-term are comparable, and the dependence of *η* on *C* changes faster than linear, yet slower than quadratic. This is exactly the behavior observed by inspecting the *η*-*C* profiles in Figure 2, which also shows that PMO length had little impact on the linear viscosity behavior at concentrations up to 30 mg/mL and that *η* vs. *C* dependence is strongly non-linear above 60 mg/mL, especially for the 25-mer and 30-mer PMOs (Figures 2B and 2C). Hence, for considerations of solution viscosity, PMO length and sequence composition become important at higher solution concentrations.

The 22-mer, 25-mer, and 30-mer PMOs are biologically relevant polymers. In theories of polymer solutions,^33^ the quadratic *C*^2^-term represents intermolecular interactions. Therefore, we performed a non-linear fit of the quadratic Huggins equation (Equation 1) in conjunction with the PMO-monomer model (model 1; see materials and methods) to the experimental data points for PMO solution viscosity. Numerical values of the intrinsic viscosity [*η*] and Huggins constant *k*_*H*_ are accumulated in Table 1. While this monomeric model describes well the viscosity data in the dilute and semi-dilute regimes (*C*< 60 mg/mL), it fails at describing the data in the concentrated solution regime for all three PMO systems, especially at 100 mg/mL (Figure 2). Hence, the intermolecular PMO-PMO interactions become significant in the high concentration regime for all three PMO molecules studied. Next, we employed the quadratic Huggins equation in conjunction with the dimer model 2 to approximate the single data point at 100 mg/mL, in order to get a sense of how different the values of [*η*] and *k*_*H*_ for PMO monomers and dimers might be. While the dimeric model captures the 100 mg/mL data point, it comes short at describing the data in the dilute and semi-dilute regimes for all three PMO systems (Figure 2). Table 1 shows that the values of [*η*] and *k*_*H*_ for PMO monomeric and dimeric forms are, indeed, substantially different.

**Table 1:** Model parameters for viscosity data for 22-mer, 25-mer, and 30-mer PMOs

PMO	Model	[*η*], mPa×s	*k*_*H*_	*C*_*dim*_, mg/mL	*C*_*trim*_, mg/mL
22-mer	Model 1	4.5 ± 0.3	4.5 ± 0.3	–	–
	Model 2	4.8 ± 0.3	5.2 ± 0.4	–	–
	Model 3	4.4 ± 0.2/5.3 ± 0.3	4.4 ± 0.3/5.4 ± 0.3	140 ± 15	–
	Model 4	–	–	–	–
25-mer	Model 1	4.7 ± 0.2	9.9 ± 0.9	–	–
	Model 2	6.2 ± 0.2	15.0 ± 1.0	–	–
	Model 3	4.6 ± 0.2/5.8 ± 0.4	9.9 ± 0.8/10.5 ± 1.2	120 ± 17	–
	Model 4	3.9 ± 0.2/4.8 ± 0.3/9.2 ± 0.7	7.0 ± 0.5/8.6 ± 0.6/16.2 ± 1.8	160 ± 17	240 ± 23
30-mer	Model 1	6.2 ± 0.3	3.8 ± 0.5	–	–
	Model 2	7.0 ± 0.4	6.1 ± 0.4	–	–
	Model 3	6.2 ± 0.3/6.8 ± 0.3	3.8 ± 0.4/4.7 ± 0.5	140 ± 14	–
	Model 4	5.3 ± 0.2/6.1 ± 0.3/6.8 ± 0.4	3.4 ± 0.2/5.1 ± 0.4/7.6 ± 0.7	190 ± 15	270 ± 25

Shown for each PMO system are the following ensemble average quantities with standard deviations: the intrinsic viscosity [η] the Huggins constant kH, and the characteristic concentrations *C*_*dim*_ and *C*_*trim*_ at which PMO dimerization and trimerization (for 25-mer and 30-mer PMOs) are nearly complete. Models 1–4 are described in materials and methods. In short, models 1 and 2 assume that PMOs are in the single-species monomeric and dimeric forms, respectively; in models 3 and 4, PMOs are allowed to form dimers, and dimers and trimers, respectively. The values of parameters for models 1–3 were obtained using the quadratic form of the Huggins equation (Equation 1); values of parameters for model 4 were obtained using the cubic form of the Huggins equation (Equation 2). Separated by the slash for model 3 are the values of [η] and kH for PMO monomers and dimers: [η]_*mon*_/[η]_*dim*_ and *k*_*H,mon*_/*k*_*H,dim*_; and for model 4 are the values of [η] and *k*_*H*_ for PMO monomers, dimers and trimers: [η]_*mon*_/[η]_*dim*_/[η]_*trim*_ and *k*_*H,mon*_/*k*_*H,dim*_/*k*_*H,trim*_.

To provide a structural basis for these findings, we analyzed the structures of PMO aqueous solutions using computational molecular modeling. We found that at 80–100 mg/mL of the 25-mer PMO solution, the PMO molecules are separated by 2.0–2.8 nm distances, which are close to the average 2.8 nm size of 25-mer PMO molecules in solution.^27^ We observed similar results for 22-mer and 30-mer PMOs. At 80–100 mg/mL, the 2.9-nm-sized 22-mer PMO molecules are separated by 1.1–2.3 nm distances, and the 3.4-nm-sized 30-mer PMO molecules (Table 2 in^27^) are separated by 1.6–3.5 nm.^27^ Therefore, given these short separation distances, the PMO molecules in concentrated solutions might interact to form higher-order structures (i.e., dimers, trimers, etc.), which warrants further exploration of PMO-PMO interactions in aqueous solution.

**Table 2:** Average molecular properties for 22-mer, 25-mer, and 30-mer PMO dimers in concentrated solution

PMO	RMSD	*D*, nm	*R*_*g*_, nm	*N*_*BP*_	*N*_*BS*_	ΔSASA, Å^2^	Δ*E*,kcal/mol
22-mer	15.6 ± 1.3	6.4 ± 0.4	1.3 ± 0.03	1.6 ± 0.4	0.7 ± 0.2	902 ± 137	−41 ± 7
25-mer	16.0 ± 0.4	6.3 ± 0.1	1.8 ± 0.01	1.4 ± 0.2	1.0 ± 0.1	1,027 ± 53	−47 ± 3
30-mer	18.6 ± 0.6	6.7 ± 0.1	1.9 ± 0.01	2.1 ± 0.2	0.8 ± 0.1	1,134 ± 63	−46 ± 3

Shown are the following ensemble average quantities (with standard errors of the mean) determined from the solution dimers for the PMO homodimers and heterodimers: RMSD, dimer size *D*, radius of gyration *R*_*g*_, number of intermolecular base pairs *N*_*BP*_ and base stacks *N*_*BS*_, buried surface area ΔSASA, and PMO-PMO interaction energy Δ*E*. All interaction energies are estimated using the MM/GBSA method (materials and methods).

### Molecular interactions in concentrated solution of PMOs

The close proximity of PMO molecules in concentrated solutions (and, hence, short diffusion time) together with their weak propensity to form stable secondary structure (through intramolecular base pairing and base stacking), makes them able to couple intermolecularly by forming the intermolecular base pairs and base stackings and backbone contacts between “morpholino nucleobases” belonging to different molecules. Yet, there is no experimental data available on PMO intermolecular interactions in concentrated solutions. To provide the structural basis of understanding PMO intermolecular interactions, we turned to computational modeling.

In our prior work,^27^ we developed an atomic force field for PMOs, building upon the force fields available for nucleic acids.^34^^,^^35^^,^^36^ We used force field bsc0_χOL3_ with improved torsion angles^37^^,^^38^^,^^39^^,^^40^ and extended it to account for atoms not described in bsc0_χOL3_ using GAFF.^41^ This PMO force field was used here to model PMO-PMO interactions for the 22-, 25-, and 30-mer PMOs (Tables S1 and S2). For each PMO, dominant conformations I, II, and III with the largest statistical weights from our previous study^27^ were selected to construct six dimer systems: three homodimers (I-I, II-II, and III-III) and three heterodimers (I-II, I-III, and II-III) (Figure 1C). Each PMO dimer was placed in a water box with an initial intermolecular separation of 0.7–1 nm, significantly smaller than the typical PMO monomer size, to mimic the concentrated solution conditions. To ensure statistically meaningful sampling of PMO association dynamics and structural variability, 10 independent MD trajectories (2 μs each) were generated for each dimer, starting from a distinct randomized initial orientation of the two PMO monomers. This yielded 60 trajectories per PMO length, and 180 simulations in total (360 μs simulation time).

### Dynamic molecular properties of PMO dimers

**Figure 3:**
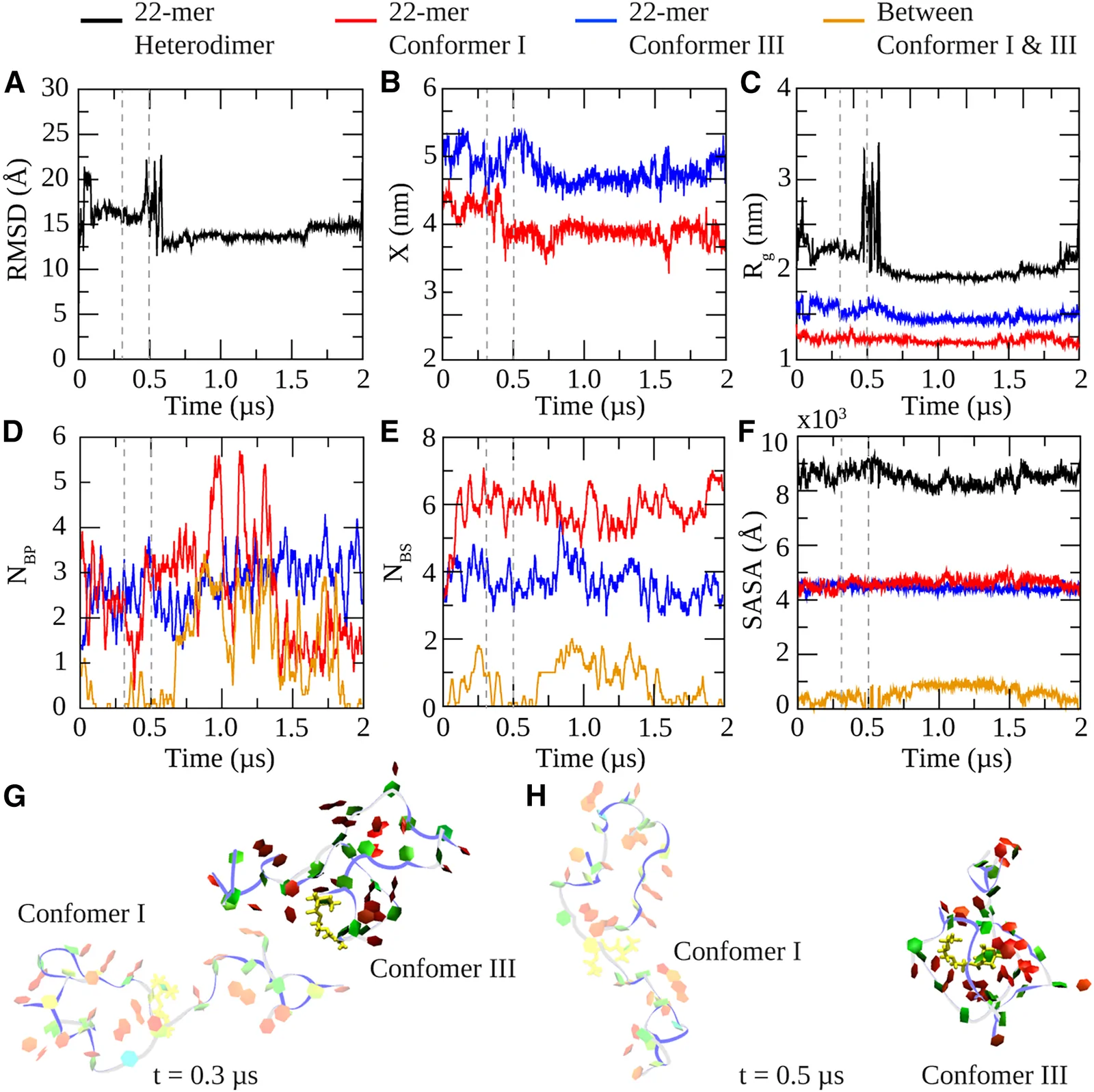
Dynamic molecular properties and snapshots for representative 22-mer heterodimer (conformers I and III) Displayed are the time profiles of the root-mean-square deviation (RMSD) (A), end-to-end distance *X* (B), radius of gyration *R*_*g*_ (C), number of base pairs *N*_*BP*_ (D), number of base stacking interactions *N*_*BS*_ (E), and solvent-accessible surface area (SASA) (F). Color denotations for molecular properties associated with the dimer, conformers I and III, and interactions between conformers I and III are explained on the graph. Also shown are structure snapshots showing interactions between conformer I (pastel color) and conformer III (non-pastel color) in paper chain and twister representation at 0.3 μs (bound state; G), and at 0.5 μs time (dissociated state; H). The PMO backbones are shown in Twister representation (blue), and nucleobases as paper chains (red and green); TEG piperazine end cap shown as yellow licorice. Dotted gray lines in (A)–(F) indicate the times at which the snapshots were taken.

**Figure 4:**
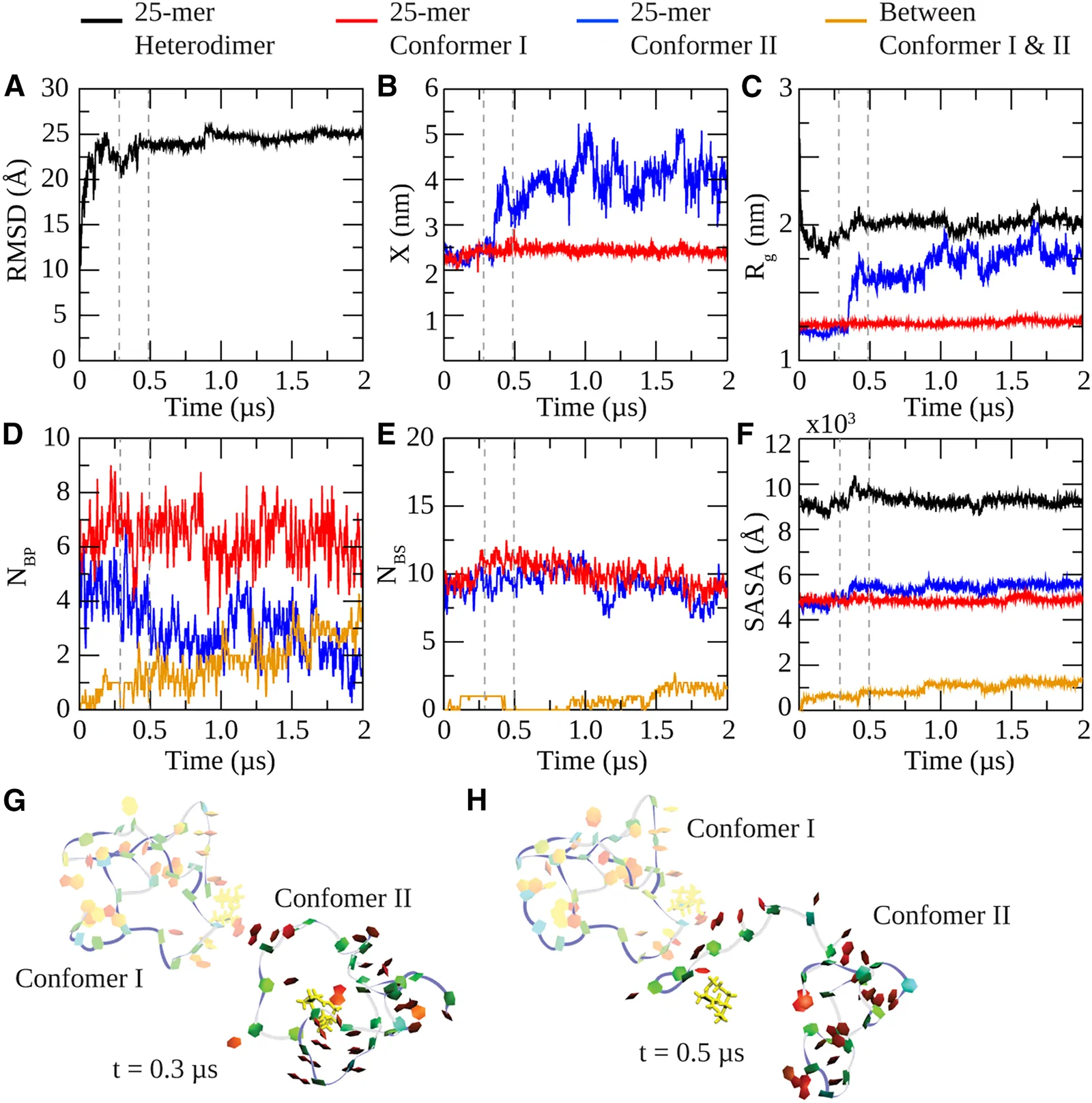
Dynamic molecular properties and snapshots for the representative 25-mer heterodimer system (conformers I and II) Displayed are the time profiles of RMSD (A), *X* (B), *R*_*g*_ (C), *N*_*BP*_ (D), *N*_*BS*_ (E), and SASA (F). Color denotations for molecular properties associated with the dimer, conformers I and II, and interactions between conformers I and II are explained on the graph. Also shown are structure snapshots showing interactions between conformer I (pastel color) and conformer II (non-pastel color) in paper chain and twister representation at 0.3 μs (bound state; G), and at 0.5 μs time (bound state; H). The PMO backbones are shown in twister representation (blue), and nucleobases as paper chains (red and green); TEG piperazine end cap shown as yellow licorice. Dotted gray lines in (A)–(F) indicate the times at which the snapshots were taken.

We analyzed the time-dependent molecular properties and show here representative examples for each PMO length that best illustrate key dynamic events: partial association, dissociation, and re-association for the 22-mer (Figure 3; Video S1), conformational opening induced by one monomer in the 25-mer (Figure 4; Video S2), and structural rearrangement in the flexible 30-mer system (Figure S2; Video S3). These time profiles provided detailed snapshots of association behavior and internal fluctuations.

Video S1. Interaction between 22-mer PMO conformers I and III during a 2-μs MD simulation at 300 K in explicit water (ice-blue transparent spheres) PMO backbones are shown in Twister representation (blue) and nucleobases as paper chains (red and green). Conformer I is shown with pastel blue backbone to distinguish it from conformer III (regular blue). The movie is 65 s long and played 4.4 × 107 times slower than computational experiment. We observe repeated association, dissociation, and re-association events between PMO molecules.

Video S2. Interaction between 25-mer PMO conformers I and II during a 2-μs MD simulation at 300 K in explicit water (ice-blue transparent spheres) PMO backbones are shown in Twister representation (blue), nucleobases as paper chains (red and green), and 5′-end TEG cap as licorice (yellow). Conformer I is shown with a pastel blue backbone to distinguish it from conformer II (blue backbone). The movie is 33 s long and played 2.2 × 10^7^ times slower than computational experiment. We observe structural rearrangement, suggesting transient and flexible interactions between PMO conformers.

Video S3. Interaction between 30-mer PMO conformers II and III during a 2-μs MD simulation at 300 K in explicit water (ice-blue transparent spheres) PMO backbones are shown in Twister representation (blue), nucleobases as paper chains (red and green), and 5′-end TEG cap as licorice (yellow). Conformer II is shown with pastel blue backbone to distinguish it from conformer III (blue backbone). The movie is 25 s long and played 1.6 × 10^7^ times slower than computational experiment. We observed unfolding and refolding of Conformer II.

PMO dimers generally form rapidly within the first 100 ns: the 22-mer PMO systems typically associate within 25–55 ns time, while the 25- and 30-mer PMO systems showed association within 30–100 ns time. Despite forming complexes, the PMO molecules remain conformationally flexible, undergoing continuous internal rearrangements and shifts in relative orientation. To monitor these conformational changes, we tracked the root-mean-square deviations (RMSDs), end-to-end distance (*X*), and radius of gyration (*R*_*g*_), which quantitate the global tertiary structure of the dimer systems. We also profiled the number of intramolecular base pairs (*N*_*BP*_) and base stacks (*N*_*BS*_), which characterize the secondary structure propensity, as well as intermolecular *N*_*BP*_ and *N*_*BS*_, which characterize the extent of PMO associations. In addition, we profiled the solvent accessible surface area (SASA) to quantitate changes in the molecular surface exposed to water solvent upon PMO-PMO interactions.

RMSD increased during the early association phase and then stabilized at ∼0.25–0.5 μs at 15–20 Å (Figures 3A, 4A, and S2A), indicating structural convergence toward a non-covalently bound dimer state. In some trajectories, RMSD stabilized much later (at ∼1 μs, data not shown), likely due to ongoing conformation changes as the dimers continue to explore alternative binding modes. Individual PMO monomers within the dimers exhibited end-to-end distance *X* fluctuations between 2 and 5 nm, occurring either in both molecules simultaneously (Figure 3B) or predominantly in one while the other remained relatively stable (Figures 4B and S2B). These fluctuations, larger than previously reported monomer values of 1.6 ± 0.5 nm (22-mer PMO), 3.2 ± 1.0 nm (25-mer PMO), and 2.8 ± 0.9 nm (30-mer PMO),^27^ likely facilitate structural adjustments that optimize intermolecular contacts, consistent with the observed RMSD behavior. Complementing RMSD and tertiary structural measures, we examined radius of gyration *R*_*g*_ as an indicator of overall molecular compactness. *R*_*g*_ of dimers ranged between 1.8 and 2.3 nm (Figures 3C, 4C, and S2C), which is less than twice the typical monomeric *R*_*g*_ of 1.2–1.5 nm observed within the dimer. When compared with previously published monomer data in dilute solution,^27^ where monomer *R*_*g*_ values were 1.5 ± 0.2 nm (22-mer PMO), 1.4 ± 0.1 nm (25-mer PMO), and 1.7 ± 0.1 nm (30-mer PMO), these findings indicate that upon dimerization, PMOs adopt more compact conformations.

The intramolecular *N*_*BP*_ ranged from 1 to 9 (Figures 3D, 4D, and S2D), while intramolecular *N*_*BS*_ (Figures 3E, 4E, and S2E) ranged from 2 to 12 for all PMOs considered. These intramolecular values show broader fluctuations than those previously reported for monomers in dilute solution, where intramolecular *N*_*BP*_ averaged 3.3 ± 0.9 (22-mer PMO), 5.9 ± 1.1 (25-mer PMO), and 6.4 ± 4.2 (30-mer PMO), and *N*_*BS*_ averaged 6.5 ± 3.6 (22-mer PMO), 8.3 ± 3.2 (25-mer PMO), and 8.7 ± 3.0 (30-mer PMO), respectively.^27^ The wider dynamic range of intramolecular *N*_*BP*_ and *N*_*BS*_ in the dimeric state suggests that dimerization increases the structural variability of each PMO as they adjust structurally to form more stable complexes. For base pairing, some monomers maintain or even enhance internal pairing, while others partially disrupt internal structure, likely to facilitate greater intermolecular associations. Similarly, stacking interactions are not uniformly maintained; they range more broadly than in the monomer state, reflecting a dynamic balance between maintaining internal structure and forming new contacts with the partner strand. This conformational plasticity indicates that PMOs do not rigidly preserve their monomeric structures upon dimerization but instead adopt diverse internal configurations to optimize intermolecular engagement. The intermolecular *N*_*BP*_ and *N*_*BS*_ each ranged from 1 to 5 for all PMOs considered (Figures 3D, 3E, 4D, 4E, S2D, and S2E), confirming that PMO dimerization is stabilized by specific base-level contacts between molecules. While base stacking continues to stabilize internal structure, base pairing appears to play a prominent role in mediating intermolecular recognition. Together, these observations point to a dynamic redistribution of base interactions: as PMOs interact, they partially rearrange their internal base pairing and stacking to accommodate new intermolecular contacts.

SASA analysis further confirmed the extent of PMO-PMO interaction upon dimerization. An area of a molecule’s surface exposed to the surrounding solvent is often correlated with molecular solubility; hydrophilic groups increase SASA, while hydrophobic regions reduce it. In the previously reported dilute PMO monomer conditions,^27^ monomer SASA ranged from ∼4,500 Å^2^ (22-mer PMO) to ∼4,800 Å^2^ (25-mer PMO), and to ∼6,000 Å^2^ (30-mer PMO). In the PMO dimer systems, monomer SASA values within the complexes were similar in magnitude, while total dimer SASAs ranged from ∼8,000 to 12,000 Å^2^ for all PMOs analyzed. To quantify the degree of surface burial upon dimerization, we computed the buried surface area, ΔSASA, defined as the difference between the total SASA of the two unbound monomers and the SASA of the dimer complex (materials and methods). By definition, ΔSASA>0 indicates that a portion of the molecular surface becomes buried in the bound state of the complex (ΔSASA = 0 implies complete dissociation). Across all systems, ΔSASA ranged from 500 to 2,100 Å^2^ (Figures 3F, 4F, and S2F), consistent with substantial and persistent intermolecular contact interfaces, representing ∼10%–25% burial relative to the monomer SASA for all three PMOs.

### Distributions of molecular properties of PMO dimers

To complement the trajectory-specific analyses described earlier, we next characterized the equilibrium structural properties of all PMO homo- and heterodimers. For each dimer, we calculated time-averaged molecular metrics over the final 200 ns of 10 independent 2-μs long simulations to capture the ensemble-level behavior. The resulting distributions of RMSD, dimer size (*D*)*, R*_*g*_, intermolecular *N*_*BP*_ and *N*_*BS*_, and buried surface area (ΔSASA) are summarized in Table S3. Across all systems, increasing PMO length (e.g., from 22-mer to 30-mer) is associated with a general rise in structural flexibility and interfacial interaction area. RMSD increases from 14.3 to 17.9 Å (22-mer PMO), to 15.1–16.6 Å (25-mer PMO), and to 16.7–21.2 Å (30-mer PMO). *D* varies in the range of 5.8–7.3 Å, while *R*_*g*_ spans the 1.2–2.0-nm range, with larger values typically associated with 30-mer dimers. Intermolecular *N*_*BP*_ varies in the 0.8–3.0 range, and *N*_*BS*_ varies in the 0.4–1.6 range, indicating modest base-mediated contacts. ΔSASA increases with PMO length from 691 to 1,105 Å^2^ (22-mer PMO), to 826–1,225 Å^2^ (25-mer PMO), and to 849–1,611 Å^2^ (30-mer PMO)—consistent with more extensive self-association interfaces in larger systems. The PMO-length specific information about the distributions of molecular properties of each PMO dimer system is accumulated in the supplemental information (see also Table S3).

When comparing homodimers and heterodimers within each PMO length, distinct patterns emerge. Homodimers generally exhibit broader structural variability, with either more extended or more compact conformations depending on the conformer type. For instance, the 22-mer I-I and 30-mer III-III homodimers are among the most extended (large *D* and *R*_*g*_), whereas the II-II homodimers also adopt more compact geometries in both 25-mer and 30-mer systems, despite forming extensive intermolecular contacts. In contrast, heterodimers, particularly I-III and II-III pairs, tend to form larger interfaces (higher ΔSASA) and, in some cases, increased base-pairing or stacking, as observed for the 30-mer I-III and 25-mer II-III systems (Table S3). These findings suggest that heterodimer formation can promote stronger interfacial interactions, driven by conformational complementarity rather than molecular size. The moderate average values of intermolecular *N*_*BP*_ and *N*_*BS*_ indicate that PMO dimerization is not dominated by nucleobase interactions (Table S3). Instead, our later analyses indicate that hydrophobic contacts between morpholino backbones likely act as an initial factor driving PMO self-association.

### PMO-PMO interaction patterns

**Figure 5:**
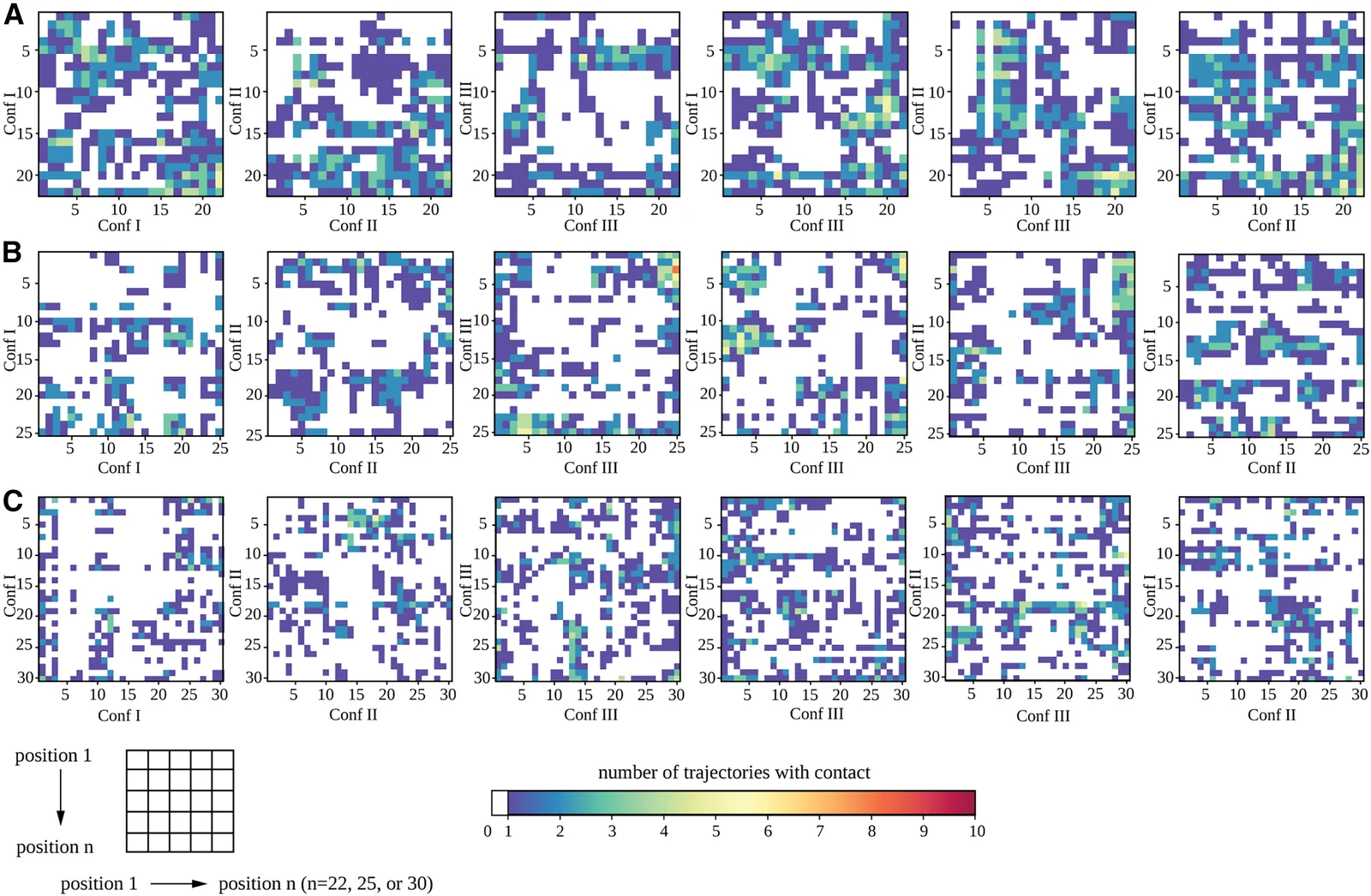
Intermolecular interaction maps of PMO-PMO dimers for 22-mer, 25-mer, and 30-mer sequences Contact maps are shown between morpholino nucleobases for the three most representative conformers (I, II, and III) of 22-mer (A), 25-mer (B), and 30-mer PMOs (C). Each map displays nucleobase-nucleobase contacts as pixels in a matrix format. The color scale indicates how many of the 10 independent simulation trajectories of a given PMO dimer exhibited a contact between each pair of positions—with a maximum value of 10 if the nucleobase pair was in contact in all trajectories. A contact is counted if the pair of nucleobases was within a defined distance threshold at any point during a given trajectory (see materials and methods). PMO bases are numbered from the 5′ end of the sequence.

To probe the sequence- and structure-specific nature of PMO-PMO interactions in dimeric assemblies, we generated contact occurrence maps for all six dimer combinations for the 22-mer, 25-mer, and 30-mer PMOs (Figure 5). Each map captures all pairwise contacts between morpholino nucleobases within and between the PMO monomers in each dimer. Each pixel represents whether an intermolecular contact between two bases was formed at least once in a trajectory. The color intensity shows in how many of 10 independent trajectories the contact was observed—ranging from 0 (white, never observed) to 1 (blue, observed in one trajectory) to 10 (red, observed in all trajectories). Thus, the maps reflect persistence of each contact across all trajectories (not its lifetime or strength).

In the 22-mer PMO dimers, 46% of all possible intermolecular contacts were never observed in any trajectory. In the 25-mer PMO dimers, 61% of all intermolecular contacts were never observed, and this fraction increased to 65% in the 30-mer PMO dimers (empty area in images in Figure 5). Among the contacts that did form, the vast majority appeared in fewer than 4 trajectories, with contacts recurring in more than 6 trajectories being extremely rare. For 22-mer PMO dimers, there was a larger fraction of contiguous contact areas, often with some higher occurrences, than was observed for the 25-mer and 30-mer dimer pairs, where contacts were often more isolated from one another. In the 25-mer and 30-mer PMO dimers, configurations involving interaction partner III showed a slightly higher fraction of contact sites (shared across 4 trajectories), suggesting some weak positional preferences of slightly higher energies in specific dimer pairings. For example, in the 25-mer III–III PMO homodimer, the most frequently recurring contact—between base 25 (C) of one monomer and base 3 (T) of the other—was observed in 8 out of 10 trajectories (Figure 5B). Nevertheless, these sporadic patterns do not indicate robust interaction motifs. Overall, the data emphasize that PMO-PMO contacts are largely transient, highly variable, and structurally heterogeneous, with no consistently recurring interface across replicates or sequence positions.

To complement this analysis, we also evaluated contact maps constructed based on a different metric: the average persistence of each contact, measured as the fraction of time a contact was present, averaged across all 10 trajectories per PMO dimer. This time-based perspective revealed similar trends (Figure S3). Consistent with the occurrence analysis, 46%, 61%, and 65% of all possible intermolecular contacts in the 22-mer, 25-mer, and 30-mer PMO dimers, respectively, were never observed. Of those that did occur, 52% (22-mer PMO), 38% (25-mer PMO), and 34% (30-mer PMO) of contacts persisted for less than 10% of the simulation time (200 ns), while only 2%, 1%, and 4% of contacts, respectively, persisted for 10%–20% of the time (200–400 ns). These data support the notion that PMO dimerization is mediated by dynamic, short-lived, and non-specific intermolecular contacts, exhibiting only weak preferential functional group interaction patterns. We acknowledge that these absolute percentages are sensitive to the chosen distance threshold; however, when using 5 Å (Figure S4) and 10 Å (Figure S5) distance cutoffs, we observed that the qualitative nature of these interactions remains unchanged, namely a lack of recurring interface interaction motifs across all dimer systems.

### Energetics of noncovalent interactions of PMO dimers

To investigate the thermodynamic basis of PMO associations in concentrated solution, we analyzed the interaction energy (Δ*E*) using the MM/GBSA method. This approach provides a means to estimate the interaction energy based on the results of all-atom MD simulations. For each 22-mer, 25-mer, and 30-mer PMO dimer system, we computed Δ*E* over the course of 2-μs trajectories, focusing on the last 200 ns (1.8–2.0 μs) for calculation of the time-averaged values (Table S3). We also calculated the ensemble average interaction energy for each PMO length (Table 2).

Among the 22-mer PMO dimers, Δ*E* ranges from −32 ± 7 kcal/mol (conformers II-II system) to −50 ± 9 kcal/mol (conformers I-I system). The 25-mer PMO dimers show a wider range, from −36 ± 6 kcal/mol (conformers I-II) up to −58 ± 6 kcal/mol (conformers II-III). For 30-mer PMO dimers, the range extends from −39 ± 5 kcal/mol (conformers I-II) to −67 ± 8 kcal/mol (conformers I-III). When averaged across all PMO dimer types, Δ*E* becomes modestly more favorable with increasing PMO length: from −41 ± 7 kcal/mol (22-mer PMO dimers), to −47 ± 3 kcal/mol (25-mer PMO dimers), and to −46 ± 3 kcal/mol (30-mer PMO dimers) (Table 2). The similarity between 25-mer and 30-mer PMO dimer values suggests diminishing enthalpic gains beyond a certain oligomer length, possibly due to dynamic conformational limits in the monomers and/or saturation of contact interfaces.

**Figure 6:**
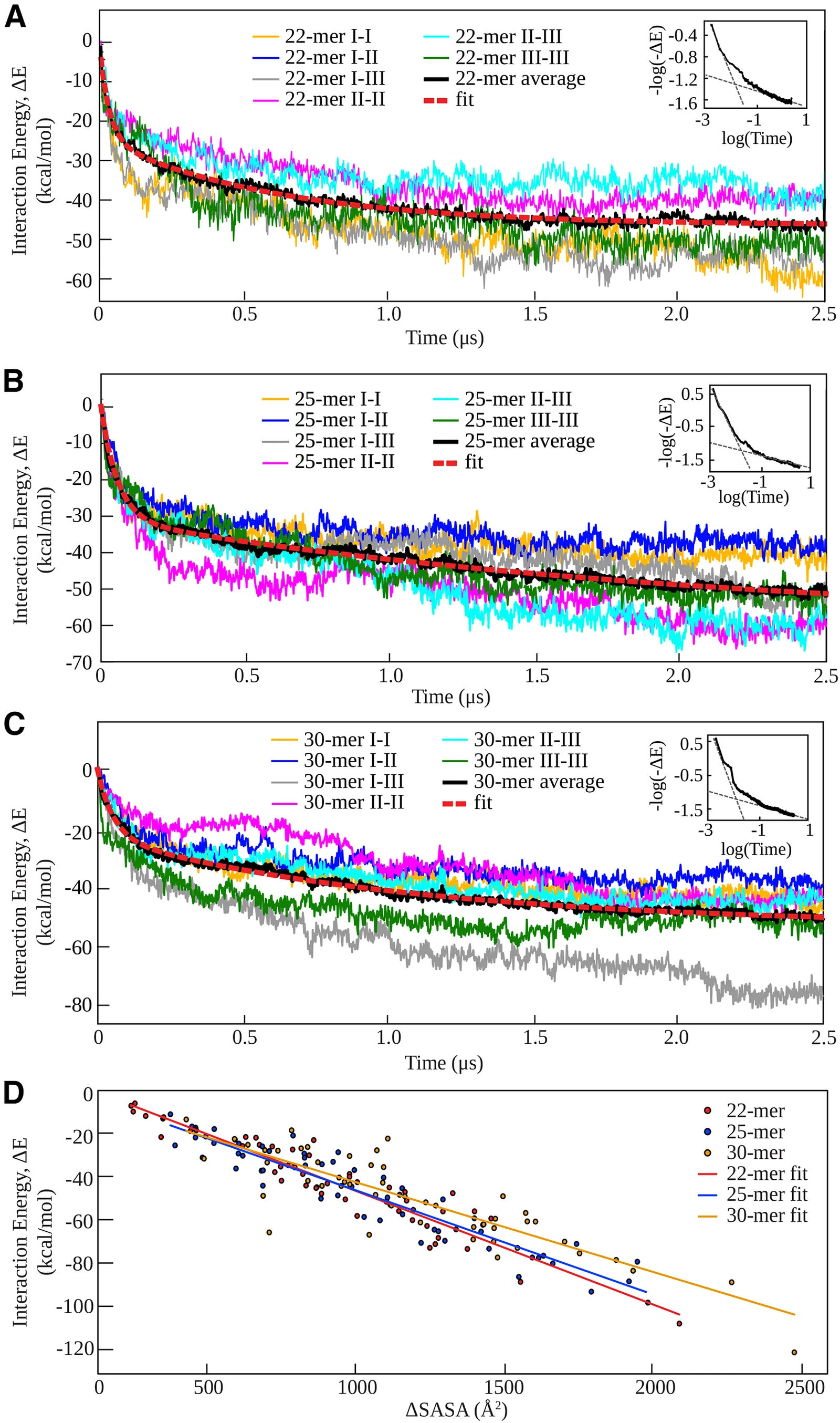
Interaction energy and correlation between interaction energies and buried surface area for PMO dimers of the 22-mer, 25-mer, and 30-mer (A)–(C) Eight trajectories of Δ*E* vs. time: orange (conformers I–I), blue (I–II), gray (I–III), magenta (II–II), cyan (II–III), green (III–III), black (overall average), and red (double-exponential fit). The insets in (A)–(C) present the log–log plot of Δ*E* versus time. (D) Scatterplots of time-averaged Δ*E* vs. ΔSASA with red, blue, and orange points representing the 22-mer, 25-mer, and 30-mer dimers, respectively; solid lines indicate linear regression fits. All interaction energy values are estimated using MM/GBSA approach and the output from simulations (materials and methods).

To quantitatively resolve the time-evolution of these intermolecular interactions and ensure interaction energy convergence for a robust analysis of the PMO interaction kinetics, we extended the trajectory analysis to 2.5 μs (Figures 6A–6C). Each image in Figures 6A–6C displays eight interaction energy curves for six individual dimer types and the average Δ*E* profile across all six dimers. At the start of the simulation (*t* = 0), Δ*E* is near zero, consistent with unassociated PMO monomers. As the PMO associate, Δ*E* decreases, indicating the formation of favorable non-covalent interactions. In most PMO dimers, this transition occurs rapidly, within the first 0.1 μs, and stabilizes around −25 kcal/mol for the 22-mer PMO, and −30 kcal/mol for the 25-mer and 30-mer PMOs. The timing and magnitude of these transitions reflect distinct binding pathways and strengths that vary with conformer pairing. Notably, the Δ*E* trajectories also exhibit a two-phase behavior: an initial fast drop followed by a slower stabilization phase (discussed in detail later).

## Discussion

PMOs are increasingly important to ASO therapeutics, with several PMO-based drugs approved for commercial use and many are in clinical trials.^28^ However, the structure-function relationships for PMOs and other ASO-based RNA mimics remain elusive because of the lack of information about their sequence-dependent solution structures. There are no X-ray crystallography data or NMR structures of PMO molecules available to date. 1D and 2D NMR techniques offer only low-resolution structural insights and are challenging to apply to large PMO molecules. For example, the 22-mer PMO molecule contains 933 atoms. Other experimental techniques such as SAXS provide information on the overall shape, conformation, and assembly state of macromolecules, but, again, they yield only low-resolution structural information. Neither NMR nor SAXS can be used to probe the time-dependent PMO interactions at the atomic level. X-ray crystallography carries information about the atomic structure, but these structures may or may not exist in aqueous solution. Limited understanding of PMO solution structures poses challenges for advancing PMO-based applications that rely on rational drug design approaches.

To overcome these limitations, we carried out computational molecular modeling of PMOs of increasing length (22-mer, 25-mer, and 30-mer) using the all-atom MD simulations under concentrated solution conditions (Figure 1). Computational modeling has long provided insights into RNA and RNA-mimic structure and dynamics.^26^^,^^27^^,^^29^^,^^42^^,^^43^^,^^44^^,^^45^^,^^46^^,^^47^^,^^48^^,^^49^^,^^50^ In our previous study of PMOs in solution,^27^ we validated our computational modeling approach by demonstrating excellent agreement between the experimental and theoretical CD spectra and solution viscosity vs. concentration curves for the 22-mer, 25-mer, and 30-mer PMOs. More recently, we extended this modeling framework to the description of the peptide-conjugated PMOs (PPMOs),^29^ and to PMOs interacting with mild surfactants.^30^ Building on these foundations, the current study used previously resolved PMO monomer structures,^27^ the three most prevalent structures in the ensemble of solution structures for each PMO, to systematically explore PMO dimerization (Figure 1). This is the first comprehensive study of PMO-PMO interactions at the atomic level of detail. Experimental viscosity-concentration profiles (Figure 2) suggest that self-association of PMOs occurs in concentrated solution, but the structures, molecular properties, and thermodynamic bases of PMO self-association remain unexplored. Yet, these insights are critical for developing PMO-based drug formulations and advancing the structure-based design of these RNA mimics. We further discuss our key findings.

### PMO-PMO interactions are dynamic and occur through broad self-association interfaces

Our prior work^27^ showed that the 22-mer, 25-mer, and 30-mer PMOs exhibit rapidly interconverting conformational subpopulations. These arise from competition between base groups and backbone moieties (phosphorodiamidate and morpholine) for solvent exposure. As a result, PMOs exist as ensembles of conformers, and this structural heterogeneity drives their tendency to self-associate in aqueous solution. This structural diversity is reflected in the broad range of the buried area of PMO dimers (ΔSASA) observed across different dimer configurations (Table S3). The buried surface areas of PMO dimers range from ∼1,000 to 2,500 Å^2^, comparable in magnitude with interfaces in protein-protein complexes, which typically bury 1,600 ± 400 Å^2^ per complex.^51^ This highlights the dynamic and extended nature of the interaction interface in PMO dimers, as opposed to localized, rigid binding pockets (e.g., knob-hole interactions in fibrin^52^) that have evolved in many biomolecular systems undergoing specific pairwise interactions.

To quantify how the interface size relates to the interaction strength, we profiled for each PMO dimer system and for each PMO length, ΔSASA as a function of MM/GBSA-derived interaction energy Δ*E* (Figure 6D), Δ*E* = *m*ΔSASA + *b*, where *m* is the slope and *b* is the *y*-intercept. Across all PMO dimers, stronger interaction energies (i.e., larger negative values of Δ*E*) are associated with greater surface burial from solvent at the interface (larger values of ΔSASA). By performing linear regression, we obtained *m* = −0.0529, *b* = 6.443 (*R*^2^ = 0.8949, *p* = 1.27 × 10^−25^) for the 22-mer PMO dimers; *m* = −0.0485, *b* = 2.3378 (*R*^2^ = 0.891, *p* = 1.23 × 10^−27^) for the 25-mer PMO dimers; *m* = −0.0418, *b* = −0.6529 (*R*^2^ = 0.751, *p* = 1.27 × 10^−17^) for the 30-mer PMO dimers. These results confirm that, with overwhelming statistical significance, larger self-association interface areas correspond to more favorable interaction energies across all PMO lengths.

Importantly, these regressions also provide a measure of how efficiently different PMO systems convert surface burial into energetic gain. Comparable correlations between buried surface area and binding free energy have been reported for protein-protein and antigen-antibody complexes.^53^^,^^54^ The 22-mer PMO dimer system exhibits the steepest slope (−0.0529 kcal/mol/Å^2^), indicating more efficient energy gain from interface formation compared to the 25-mer (−0.0485 kcal/mol/Å^2^) or 30-mer systems (−0.0418 kcal/mol/Å^2^). This trend suggests that shorter PMOs achieve stronger stabilization per unit of contact area, likely due to tighter packing and reduced conformational flexibility, which allows more effective energetic coupling across the interface. In contrast, longer PMOs distribute interactions over broader and more adaptable surfaces, leading to slightly lower per-area efficiency despite forming larger overall interfaces. Such dynamic, heterogeneous self-association is also observed in intrinsically disordered proteins^55^ and flexible nucleic acids.^56^ In these systems, interaction energy generally scales with the contact area rather than being confined to rigid, localized pockets, paralleling the behavior seen for PMO dimers.

### PMO-PMO interactions are energetically favorable and involve backbone-mediated hydrophobic contacts

The broad range of interaction energies in each trajectory, i.e., −5 to −108 kcal/mol for the 22-mer PMO dimers, −11 to −98 kcal/mol for the 25-mer PMO dimers, and −13 to −122 kcal/mol for the 30-mer PMO dimers (Figure 6D), suggests that under concentrated solution conditions, PMO-PMO interactions are energetically favorable and structure-dependent. Understanding this behavior is critical for optimizing stability and predicting PMO behavior in concentrated drug products.

When compared with monomer folding energies,^27^ the dimer interaction energies are generally comparable in magnitude. The MM/GBSA-derived values of PMO folding energy come to −44 to −75 kcal/mol for 22-mer PMO, −79 to −114 kcal/mol for 25-mer PMO, and −85 to −113 kcal/mol for 30-mer PMO. As a result, the PMO dimer interaction energies are lower than the sum of folding energies of two PMO monomers, indicating that PMO monomers largely retain their pre-folded structure upon self-association, rather than undergoing complete structural rearrangement. Indeed, while partial conformational adjustments were observed in some simulations (Video S1), complete unfolding or co-folding into a new joint structure was not observed. This suggests that PMO dimers form through surface-mediated interactions where pre-folded monomers associate via exposed regions. Furthermore, moderate PMO dimer interaction energies are reflected by the relatively low numbers of intermolecular base pairs and stacking interactions (Tables 2 and S3). The average number of intermolecular base pairings *N*_*BP*_ and base-stackings *N*_*BS*_ vary in the 1.6–2.1 range and in the 0.7–1.0 range, respectively. Such modest levels of canonical base pairing and π-π stacking interactions indicate that PMO association is not primarily driven by extensive hydrogen bonding or base stacking, as in canonical nucleic acid duplexes. Instead, our findings point to the importance of additional molecular forces.

Polar interactions between complementary atom groups and hydrophobic contacts among the relatively nonpolar morpholino rings and phosphorodiamidate backbone regions likely contribute to dimer stability. Selected time-averaged numbers of hydrophobic contacts from MD trajectories for the 22-mer PMO dimers (Figure S6B), 25-mer PMO dimers (Figure S7B), and 30-mer PMO dimers (Figures S8B and S9B), show that these interactions form early and persist locally, supporting their stabilizing role. To ensure this interpretation was not dependent on the specific 7.5 Å distance threshold used, we re-analyzed the data using the 5 Å (Figures S10–S12) and 10 Å (Figures S13–S15) cutoffs. While the absolute number of hydrophobic contacts changed with the variable distance cutoff, the early onset and relative distribution of contacts across the backbone remained consistent, confirming that hydrophobic stabilization is a robust feature of PMO self-association. This interpretation is consistent with literature findings on synthetic nucleic acid analogs. For example, phosphorodiamidate piperidino oligomers (PPOs), which differ from PMOs in having more hydrophobic piperidino residues, exhibit enhanced lipophilicity and self-association compared to PMOs of identical sequence.^57^ Similarly, phosphorothioate (PS)-modified oligonucleotides engage in hydrophobic interactions through their sulfur-containing backbones, which contribute to increased affinity for proteins and cellular uptake.^58^ More broadly, the significance of hydrophobic interactions as fundamental drivers of self-assembly in biomolecules, including nucleic acids, has been widely recognized.^59^^,^^60^ In concentrated environments, both entropic and enthalpic effects—particularly hydrophobic association—play crucial roles in mediating molecular interactions.^61^ Collectively, these principles support our observations that PMO-PMO association arises not only from canonical base-pairing but from a combination of hydrophobic and polar interactions that stabilize PMO assemblies.

### PMO-PMO interactions are non-specific and do not involve preferred interaction sites

The broadly distributed and variable nature of PMO-PMO contacts underscores the non-specific character of PMO-PMO interactions. Contact occurrence maps reveal low-intensity, widely dispersed interactions along the sequence, with no consistent hotspots (Figures 5 and S3). This pattern reflects the randomized initial orientations of PMO monomers, allowing productive contacts to nucleate at many locations and resulting in no dominant seed site for association. This spatial variability indicates that, for the three specific sequences tested in this study, PMO self-association does not rely on sequence-encoded recognition motifs, but instead proceeds via transient, surface-driven encounters shaped by local structural accessibility. In contrast to canonical nucleic acids, where precise base pairing and geometric complementarity produce highly reproducible binding interfaces, PMO-PMO interactions are governed more by flexible conformations, hydrophobic interactions, and transient contact opportunities.

The lack of recurring contact regions further supports this interpretation: that most of all potential intermolecular contacts never form, and those that do are rarely shared across multiple trajectories. For PMOs, this behavior is likely rooted in the presence of the uncharged, morpholino-modified backbone, which diverges significantly from the canonical negatively charged, base-stacking architecture of RNA and DNA. The base-base contact persistence analyses (Figure S3) reinforce this view. Many base-base contacts observed during early association are not retained over time, highlighting their provisional and exploratory nature. Instead, hydrophobic interactions, i.e., those involving nonpolar morpholino rings and linker regions, form early and tend to persist throughout the simulation, acting as stabilizing anchors. These contacts are less sensitive to precise alignment and more tolerant of conformational variation, making them key contributors to interface retention. Altogether, these findings highlight the fact that PMO-PMO interactions follow a fundamentally different mode of self-association than canonical nucleic acids. Their reliance on non-specific, shape-driven interactions likely influences how PMOs behave in high-concentration formulations, where such transient associations could trigger PMO aggregation and affect intracellular distribution.

### PMO-PMO interaction occurs via fast anchoring followed by a slow binding contact optimization mechanism

The time profiles of the average PMO-PMO interaction energies for PMO dimers reveal a biphasic self-association process characterized by two distinct slopes—which are clearly observed on a log-log plot (Figures 6A–6C, insets). The initial steep energy drop in the first ∼100 ns reflects rapid interaction events, followed by a much slower and more gradual decline extending from 0.1 to 2.5 μs. These observations suggest that PMO association proceeds through two different association mechanisms. To quantitatively capture the corresponding timescales and energy gains, we performed a fit of the average interaction energy curves to a double-exponential model, $\Delta E\left(t\right)=-\Delta E+{\Delta E}_{1}e^{-t/\tau_{1}}+\left(\Delta E-\Delta E_{1}\right)e^{-t/\tau_{2}}$, with *τ*_1_ and *τ*_2_ representing the characteristic timescales and Δ*E*_1_ and Δ*E*_2_ = Δ*E*-Δ*E*_1_ being the energy contributions from the fast and slow binding modes, respectively (Δ*E* is the total interaction energy). For the 22-mer dimer, *τ*_1_ = 33.0 ± 2.20 ns, *τ*_2_ = 0.61 ± 0.02 μs, Δ*E*_1_ = 22.8 ± 0.38 kcal/mol, and Δ*E*_2_ = 22 ± 0.15 kcal/mol (Δ*E* = 44.0 ± 0.16 kcal/mol; *p* < 10^−308^). For the 25-mer PMO dimer, *τ*_1_ = 57.2 ± 1.90 ns, *τ*_2_ = 2.1 ± 0.19 μs, Δ*E*_1_ = 29.1 ± 1.19 kcal/mol, and Δ*E*_2_ = 30.3 ± 1.21 kcal/mol (Δ*E* = 59.4 ± 1.43 kcal/mol; *p* < 10^−308^). For the 30-mer PMO dimer, *τ*_1_ = 54.0 ± 2.70 ns, *τ*_2_ = 1.08 ± 0.04 μs, Δ*E*_1_ = 30.8 ± 0.31 kcal/mol, and Δ*E*_2_ = 33.2 ± 0.36 kcal/mol (Δ*E* = 54.0 ± 0.41 kcal/mol; *p* < 10^−308^).

These results show that about half of the total interaction energy accumulates rapidly during the fast phase (*τ*_1_ = 30–60 ns), while the remaining half of the energy is gained more gradually during the subsequent microseconds. The initial, rapid association phase is largely PMO length-independent and is rate-limited by the relative diffusion of PMO monomers. Indeed, the morpholino backbone (and linker) chemistry is shared among all PMOs, and so the kinetics of this initial encounter are governed primarily by PMOs’ diffusion and local surface complementarity search. In contrast, *τ*_2_ = 0.6–2.1 μs varies more substantially across PMO dimers, reflecting the slower conformational rearrangements and optimization of binding contacts, which depend on molecular flexibility and sequence length. Subsequent structural and energetic analyses summarized later reveal, despite significant binding pattern variability, structural details underlying these two binding phases.

For some PMO dimers (e.g., 30-mer PMO conformers III–III, Figure S9), binding remains weak. Backbone-mediated hydrophobic and base contacts only begin forming after 0.2 μs, coinciding with a sharp decrease in interaction energy and a modest rise in hydrophobic interactions. However, the number of hydrophobic contacts is low (∼5), resulting in only mild interaction energies (∼-20 kcal/mol). In contrast, dimers with stronger interactions (e.g., 25-mer PMO conformers III-III, Figure S7) display a clear correlation between interaction energy and hydrophobic contacts: as interaction energy decreases, the number of backbone-mediated hydrophobic contacts increases. These hydrophobic interactions stabilized within the first 0.1 μs and continue to strengthen over time, leading ultimately to consistently strong interaction energies (∼−125 kcal/mol). Stable backbone hydrophobic and base-base anchors form rapidly and persist throughout the simulation.

These observations reveal a characteristic pattern of PMO self-association. During the initial, fast-binding phase, PMO monomers engage in transient, nonspecific interactions—primary hydrophobic contacts between morpholino backbones—which serve as anchoring points that keep the monomers in proximity. Stabilizing contacts are retained while weaker, less productive ones are lost, allowing the monomers to reorient and progressively refine their interfacial geometry and stabilization energy. This refinement enables the formation of additional base stacking, pairing, and polar interactions. This is consistent with the slower phase large-scale global structural changes as compared to the local structure change, fast complex-forming phase, where diffusion-induced non-covalent interactions dominate. More extended PMO monomers (e.g., 30-mer PMO conformers II-III, Figure S8) are particularly effective in optimizing such structural refinement, whereas compact conformers (e.g., 22-mer PMO conformers II-II, Figure S6) exhibit limited progression, underscoring the importance of conformational adaptability in PMO self-association.

### Interpretation of concentrated PMO solution viscosity data involves considering the self-association of PMO monomers

In analyzing the experimental solution viscosity data for the 22-mer, 25-mer, and 30-mer PMOs (Figure 2), we obtained the following results. The monomer model 1 comes short in the concentrated solution regime for *C*> 75 mg/mL, especially for the 25-mer and 30-mer PMOs (Figures 2B and 2C). The dimer model 2 fails at describing the data in the dilute and semi-dilute solution regimes for all three PMOs (Figure 2). We hypothesized that this discrepancy arises from self-association of PMO in concentrated solution, which the theory did not account for. Indeed, at high concentration, the molecular size (∼2*R*_*g*_) is comparable with the intermolecular distance (Δ*x*), and the average diffusion time for two PMO molecules to approach each other can be estimated as $\Delta t\approx \frac{2\pi \eta R_{g}\Delta x^{2}}{k_{B}T}$, where *k*_*B*_ is the Boltzmann’s constant (1.38 × 10^−23^ J/K), *T* is absolute temperature, and *η* is solution viscosity. Using *η* = 4.5 mPa×s (midpoint between 75 and 100 mg/mL for the 25-mer PMO; Figure 2B), *R*_*g*_ = 1.8 nm, Δ*x* = 2*R*_*g*_ = 3.6 nm (Table 2), and *T* = 300 K, we obtain the diffusion time Δ*t*≈ 160 ns. This indicates that in concentrated solutions 25-mer PMO monomers can diffuse together and start interacting within 200 ns. For the other PMOs, we obtained similar results: Δ*t*≈ 60 ns (22-mer PMO) and 190 ns (30-mer PMO).

Using computational molecular modeling of the 22-mer, 25-mer, and 30-mer PMO dimers, we have systematically explored the structural and energetic factors underlying PMO self-association, providing a theoretical basis for formation of higher-order PMO solution structures and meaningful interpretation of the solution viscosity vs. solution concentration data. Building on the intuition gained, we now revisit the experimental viscosity data and propose using dimerization model 3 (together with Equation 1 in materials and methods), which accounts for self-association of PMO monomers into dimeric species around some characteristic PMO concentration *C*_*dim*_, and trimerization model 4, together with the cubic form of the Huggins equation (Equation 2), which also takes into account self-association of PMO monomers into trimeric species around some characteristic PMO concentration *C*_*trim*_ (*C*_*dim*_ and *C*_*trim*_ should be understood as concentrations at which the predominant population of PMO molecules exists in the dimeric and trimeric states, respectively). We note that in this study, the trimeric PMO species are not directly simulated; rather, PMO trimers are considered phenomenologically in conjunction with the cubic form of Huggins equation (Equation 2) to describe the solution viscosity data. The results of applying models 3 and 4 are presented in Figure 2 (parameter values are in Table 1).

The dimerization model 3 works well in the concentrated solution of the 22-mer PMO (Figure 2A), which means that the 22-mer PMO monomers self-associate into dimers but not trimers. For the 22-mer PMO, we found the monomer and dimer intrinsic viscosities [*η*]_*mon*_ = 4.4 ± 0.2 mPa×s and [*η*]_*dim*_ = 5.3 ± 0.3 mPa×s, and Huggins constants *k*_*H*,*mon*_ = 4.4 ± 0.3 and *k*_*H*,*dim*_ = 5.4 ± 0.3 (Table 1). While theoretical curves of solution viscosity for the 25-mer and 30-mer PMOs from dimerization model 3 improve upon the performance of the monomer model 1, theoretical curves still underestimate the experimental viscosity at 100 mg/mL (Figures 2B and 2C). For this reason, we used the trimerization model 4 to describe the solution viscosity data for the 25-mer and 30-mer PMO solutions. This model reproduces experimental viscosity-concentration profiles for 25-mer and 30-mer PMO solutions (Figures 2A and 2B). For the 25-mer PMO, we obtained [*η*]_*mon*_ = 3.9 ± 0.2 mPa×s, [*η*]_*dim*_ = 4.8 ± 0.3 mPa×s, and [*η*]_*trim*_ = 9.2 ± 0.7 mPa×s, and *k*_*H*,*mon*_ = 7 ± 0.5, *k*_*H*,*dim*_ = 8.6 ± 0.6, and *k*_*H*,*trim*_ = 16.2 ± 1.8. For the 30-mer PMO, we obtained [*η*]_*mon*_ = 5.3 ± 0.2 mPa×s, [*η*]_*dim*_ = 6.1 ± 0.3 mPa×s, and [*η*]_*trim*_ = 6.8 ± 0.4 mPa×s, and *k*_*H*,*mon*_ = 3.4 ± 0.2, *k*_*H*,*dim*_ = 5.1 ± 0.4, and *k*_*H*,*trim*_ = 7.6 ± 0.7 (Table 1). We found that, according to model 3, dimerization of the 22-mer PMO is nearly complete at *C*_*dim*_ = 140 ± 15 mg/mL. According to trimerization model 4, there is substantial dimerization of the 25-mer PMO at *C*_*dim*_ = 160 ± 17 mg/mL and 30-mer PMO at *C*_*dim*_ = 190 ± 15 mg/mL, and there is substantial trimerization of the 25-mer PMO at *C*_*trim*_ = 240 ± 23 mg/mL and 30-mer PMO at *C*_*trim*_ = 270 ± 25 mg/mL.

The shape of PMO molecules, as reflected by their *D*/*R*_*g*_ ratios, provides insight into their solution behavior. The 22-mer PMO is highly rodlike, while the 25-mer and 30-mer are moderately elongated. Molecular weights may also contribute to hydrodynamic properties: the 22-mer dimer (MW = 15.7 kDa), 25-mer dimer (MW = 17.3 kDa), and 30-mer dimer (MW = 20.6 kDa) increase progressively, but the viscosity observed experimentally does not scale simply with mass. Notably, the 25-mer PMO exhibits an unexpectedly high viscosity at 100 mg/mL. This behavior likely arises from a combination of moderate rodlike geometry and a molecular surface that favors intermolecular contacts, promoting self-association into dimers and trimers. In contrast, the 22-mer PMO, despite its highly rodlike structure, has a smaller surface and fewer interaction sites, limiting higher-order assembly and resulting in lower viscosity. The 30-mer PMO, while larger, is more flexible and less elongated, which reduces the efficiency of packing and alignment for intermolecular association. Consistent with these observations, modeling using dimerization and trimerization Huggins equations shows that the 25-mer PMO forms significant amounts of dimers and trimers at concentrations above 160 mg/mL (dimer) and 240 mg/mL (trimers), whereas the 22-mer and 30-mer PMOs remain largely monomeric or form only dimers at those concentrations.

To conclude, this research represents the first systematic, atomic-level characterization of the structural, energetic, and hydrodynamic features governing biomolecular interactions of PMOs in aqueous solution. Through integrated experimental and computational analysis, including detailed MD simulation-based descriptions of the kinetics, energetics, and interacting functional groups of the pairwise PMO-PMO dimerizations, we demonstrate that different PMO conformers, in the solution ensemble of conformations, undergo self-association, initiated by hydrophobic coupling between morpholino and phosphorodiamidate backbones, subsequently stabilized by a limited number of base pairings and stackings. This type of interaction to form dimers contrasts with that of the canonical oligonucleotide charged backbone and the large electrostatic interactions that govern their intermolecular associations. These findings refine our molecular understanding of PMO behavior under pharmaceutically relevant conditions and inform strategies for formulation development and storage conditions for future therapeutic design.

Our analysis of the MD simulations of pairwise combinations of the most prevalent conformers of each of the 22-mer, 25-mer, and 30-mer PMOs has identified a number of critical features of PMO-PMO interactions that have direct implications for therapeutic development. First, PMO conformers dimerize through extended, heterogeneous interaction surfaces (1,000–2,500 Å^2^ buried surface area), rather than localized binding pockets, representing 10%–25% of monomer surface area. Second, dimerization enthalpies range from −5 to −108 kcal/mol for the 22-mer PMO, from −11 to −98 kcal/mol for the 25-mer PMO, and from −13 to −122 kcal/mol for the 30-mer PMO (these values indicate that longer PMOs show incrementally enhanced self-association). Third, for all three PMO dimers studied, stronger interaction energies are associated with greater surface burial (larger values of buried surface area). Since the slope of the regression line represents the average energetic contribution per Å^2^ of buried surface area–effectively, the “binding efficiency” of the interface,^51^ we find that the 22-mer PMO dimer system exhibits the most efficient energy gain from interface formation (*m* = −0.0529), followed by the 25-mer (*m* = −0.0485), then the 30-mer (*m* = −0.0418). Fourth, ∼61–65% of all possible PMO position-position intermolecular interactions never form; those interactions that do form appear in fewer than four out of ten trajectories of the same system, indicating that dimerization involves at best only weakly preferred transient interactions at given positions in the dimer pair. Fifth, the dimerization dynamics for each PMO exhibits two-phase kinetic behavior, a rapid intermolecular “anchoring” phase (*τ*_1_≈ 30–60 ns), driven by close to diffusion-limited formation of backbone hydrophobic contacts, followed by a slow energy optimization phase (*τ*_2_≈ 0.6–2.1 μs) involving a degree of conformational flexibility in the interacting monomers and evolution of the interaction pattern to produce a small level of intermolecular base pairing and base stacking. Interestingly, the fast-phase kinetics are PMO length-independent, while the slow-phase kinetics depends on molecular flexibility.

Based on the results of the all-atom MD simulations of PMO dimerization, the experimental viscosity data could be explained, since the dimerization simulations were carried out at the PMO concentrations in the viscosity experiments where non-linear viscosity increases occur. While trimer formation was not directly studied in MD simulations, it could be inferred from fitting experimental viscosity–concentration data using higher-order PMO association models. It was found that the 22-mer PMO monomers self-associate into dimers but not trimers, while the 25-mer and 30-mer PMOs undergo dimerization and then formation of higher-order structures. These studies add to the small but growing body of knowledge about PMOs and their properties, and it is relevant to issues related to PMO formulation and dosage development for a new generation of PMO therapeutics. Future work will focus on direct thermodynamic and biophysical validation of the predicted PMO self-association energetics using complementary experimental approaches such as isothermal titration calorimetry, differential scanning calorimetry, and solution-phase aggregation measurements.

## Materials and methods

### Viscosity-concentration data

Experimental viscosity versus concentration data were collected for the 22-mer, 25-mer, and 30-mer PMOs as described in detail in our previous publications.^27^^,^^29^

### Constructing PMO dimer systems

Atomic partial charges for each 22-mer, 25-mer, and 30-mer PMO system and force field parameters for MD simulations of the PMO-PMO interactions are described in our previous publications,^27^^,^^29^ and are summarized in supplemental information. The atomic structural models of PMOs comprised of 22 nucleobases (22-mer), 25 nucleobases (25-mer), and 30 nucleobases (30-mer) were constructed as previously described.^27^ For each PMO system, we selected the atomic structures of the top three conformations I, II, and III with the highest statistical weight, identified previously.^27^ By pairing the three conformers in different combinations, we constructed six PMO dimer systems: three homodimer systems for conformer I, conformer II, and conformer III, as well as 3 heterodimer systems for conformers I and II, I and III, and II and III. In the initial structures, we placed two PMO monomers 7–10 Å apart. In the MD simulations, we opted not to model relative diffusion of PMO molecules and focused on PMO association events. For this reason, the initial 0.7–1.0-nm separation was chosen to probe only the kinetics of PMO-PMO interaction following molecular encounter. The larger separation distances would have resulted in microsecond-scale diffusion (before PMO-PMO interactions set in), which is outside the focus of this study. For each PMO dimer system, we carried out 10 independent MD simulation runs, each with a different relative PMO monomer orientation to mimic their randomness in solution, each run being 2-μs long. This is a total of 6 × 10 = 60 runs per PMO system and 60 × 3 = 180 simulation runs for all PMOs.

### MD simulations

The all-atom MD simulations were carried out as described in previous studies.^27^^,^^29^^,^^30^ Briefly, each PMO-PMO dimer system (i.e., heterodimers formed by conformers I and II, conformers I and III, and conformers II and III and homodimers formed by conformers I and I, conformers II and II, and conformers III and III) and for each 22-mer, 25-mer, and 30-mer PMO, was solvated in a rectangular water box, containing two corresponding PMO molecules wrapped with water molecules. Depending on conformer types and PMO system used, the solvation boxes contained ∼9,600–20,300 water molecules for the 22-mer PMO-PMO systems (which corresponds to 413–778 nm^3^ solvation box volume range), ∼10,500–19,900 water molecules for the 25-mer PMO-PMO systems (∼424–726 nm^3^ solvation box volume range), and ∼13,500–29,900 water molecules (∼533–1,053 nm^3^ solvation box volume range) for 30-mer PMO-PMO systems. These conditions correspond to an effective PMO concentration of ∼4–8 mM. PMOs are uncharged molecules, so counterions were not included. Each PMO-PMO system was energy minimized using the steepest descent algorithm^62^ over 50,000 steps and then using the conjugate gradient method over 5,000 steps; the 50 kcal/mol energy restraint was applied to all solute atoms. Gradual heating of each system was carried out from 0 to 300 K (target temperature) during 50-ps time. Next, 200 ps of restrained MD simulations (with 0.05 kcal/mol energy constraint placed on all solute atoms) were performed to equilibrate each system. The 2-μs-long unrestrained production MD simulations in water at 300 K were carried out for each PMO-PMO system using the CUDA version of pmemd^63^ in the GPU-accelerated^62^^,^^64^ AMBER 20 package^65^ (Figure 1C). The output from MD simulations (Figure 1D) was analyzed as described later.

### Analysis of molecular dynamics simulations

The RMSDs for all atoms of PMO molecules and their end-to-end distances (*X*) were calculated using the VMD package as previously described.^42^ For RMSD analysis, all the frames were superimposed with the initial energy-minimized structure of the PMO dimer system in question (in unassociated state) after removing water molecules. *X* was calculated as the distance between the first and last P-atom of each PMO monomer in each dimer system. The radius of gyration *R*_*g*_ was calculated using the formula, $R_{g}={\left(\sum_{q}^{0}m_{p}r_{p}^{2}/\sum_{q}^{0}m_{p}\right)}^{1/2}$, where *m*_*p*_ and *r*_*p*_ are the mass and position of atom *p* relative to the center of mass of the molecule. The SASA was estimated using the LCPO algorithm^66^ implemented in the CPPTRAJ module^67^ in AmberTools20.^65^ The buried surface area associated with formation of the PMO dimer, ΔSASA, was calculated as ΔSASA = SASA_*mon*1_ + SASA_*mon*2_ − SASA_*dim*_, where SASA_*mon*1_ and SASA_*mon*2_ are SASA*s* for monomers 1 and 2, respectively, as if they were in unassociated states, and SASA_*dim*_ is the dimer SASA. The total number of base pairs, *N*_*BP*_, and the number of base stackings, *N*_*BS*_, were calculated using Barnaba software^68^ (see Figure S2). To identify PMO-PMO interaction contacts, we assumed that a pair of nucleotides have interactions (electrostatic, hydrophobic, hydrogen bonds, etc.) if the distance between the C5 atoms of the bases is <7.5 Å-cutoff distance (see supplemental information). To verify that the mechanistic conclusions are not biased by this specific distance threshold, we repeated the analysis with alternate distance cutoffs of 5 and 10 Å. To obtain the distribution of distances between the PMO molecules, we used the MDAnalysis Python package.^69^^,^^70^ The complex size *D* was defined as the largest pairwise distance observed between atoms in one monomer and in the other monomer in the dimer. The average values of RMSD, *D*, *R*_*g*_, *N*_*BP*_, *N*_*BS*_, ΔSASA, and Δ*E* (Table 2) were obtained by calculating the time-averages over the last 1.8–2-μs portion of trajectories simulated.

### Thermodynamic state functions

The interaction energy (enthalpy) Δ*E* between PMO strands for each 22-mer, 25-mer, and 30-mer PMO dimer system was computed using the molecular mechanics/generalized Born surface area (MM/GBSA) method as implemented in MMGBSA.py program from the AMBER suite.^71^^,^^72^ The MM/GBSA-based calculations were performed using the following settings. The polar solvation energy was estimated using the generalized Born model (with the ionic strength of the solvent set to 0 M). For each analyzed frame, the energies of the dimer and monomers were computed separately; the interaction energy Δ*E*, which represents the interaction enthalpy rather than the binding free energy, was calculated as: Δ*E* = *E*_*dim*_ − *E*_*mon*1_ − *E*_*mon*2_, where *E*_*dim*_, *E*_*mon*1_, and *E*_*mon*2_ are the energies of the dimer, one monomer, and the other monomer, respectively. Each energy term consists of molecular mechanical and solvation energy components: *E* = *E*_*int*_ + *E*_*C*_ + *E*_*vdW*_ + *E*_*p*_ + *E*_*np*_, where *E*_*int*_ includes bonded energies (for bonds, bond angles, and dihedral angles), *E*_*C*_ is the Coulombic energy, *E*_*vdW*_ is the van der Waals energy, and *E*_*p*_ and *E*_*np*_ are the polar and non-polar solvation energies, respectively. The polar contribution was estimated via the GB model, whereas the non-polar contribution was computed from the solvent-accessible surface area (SASA).^73^

### Reconstruction of PMO solution viscosity vs. concentration profiles

Our approach to the theoretical reconstruction of the viscosity (*η*) vs. concentration (*C*) profiles is described in detail in our prior studies.^27^^,^^29^ In this study, we applied several models of PMO solution viscosity to describe the experimental viscosity-concentration behavior. The perturbative treatment of the dependence of viscosity of polymer solutions on polymer concentration is given by the Huggins quadratic equation,

(Equation 1) $$\eta =\eta_{0}\left(1+\left[\eta \right]C+k_{H}{\left[\eta \right]}^{2}C^{2}\right)$$

and cubic equation,

(Equation 2) $$\eta =\eta_{0}\left(1+\left[\eta \right]C+k_{H}{\left[\eta \right]}^{2}C^{2}+\frac{k_{H}^{2}}{2}{\left[\eta \right]}^{3}C^{3}\right)$$

where *η*_0_ is viscosity of water, [*η*] is the average intrinsic viscosity, and *k*_*H*_ is the Huggins constant. The non-linear Huggins Equation 1 and 2 are invoked to describe interactions between polymer chains in the concentrated solution regime (when the distance between molecules becomes comparable with their size). Importantly, all the MD simulations performed in this work are limited to monomeric and dimeric PMO systems; no trimer or higher-order PMO aggregates were directly simulated. Any discussion of PMO trimers arises solely from interpretation of the viscosity-concentration data with help from models 1–4 described later. The standard deviations for the model parameters from the fit of models 1–4 to the viscosity vs. concentration profiles were evaluated using the experimental standard deviations, which are ∼10% of the viscosity values due to temperature-related measurement variations.

#### Model 1

PMO molecules exist in solution as monomers that weakly interact with each other, but without the formation of PMO dimers, and the viscosity-concentration data are described by the quadratic Huggins Equation 1 with [*η*] and *k*_*H*_ being the intrinsic viscosity and Huggins constants for PMO monomers.

#### Model 2

PMOs are dimers, but formation of higher-order forms (trimers, etc.) does not occur. The viscosity-concentration data are described by the quadratic Huggins Equation 1 with [*η*] and *k*_*H*_ being the intrinsic viscosity and Huggins constants for PMO dimers.

#### Model 3

PMOs are monomers at low concentrations, forming stable dimers in the intermediate-to-high concentration regime. The viscosity-concentration data are described by the quadratic Huggins Equation 1 with the average [*η*] = *p*_*mom*_[*η*]_*mom*_ + *p*_*dim*_[*η*]_*dim*_ and average *k*_*H*_ = *p*_*mom*_*k*_*H*,*mon*_ + *p*_*dim*_*k*_*H*,*dim*_ given by the weighted sum of contributions from the monomeric form ([*η*]_*mon*_ and *k*_*H*,*mon*_) and dimeric form ([*η*]_*dim*_ and *k*_*H*,*dim*_) with the monomer weight $p_{mon}=e^{-C/C_{\dim}}$ decreasing and dimer weight $p_{\dim}=1-p_{mon}=1-e^{-C/C_{\dim}}$ increasing with concentration *C* over some characteristic concentration for dimerization *C*_*dim*_.

#### Model 4

PMOs exist as monomers at low concentrations that form stable dimers in the intermediate-to-high concentration regime; dimers form trimers in the high-concentration regime. The data are described by the cubic Huggins Equation 2 with the average [*η*] = *p*_*mon*_[*η*]_*mon*_ + *p*_*dim*_[*η*]_*dim*_ + *p*_*trim*_[*η*]_*trim*_ and average *k*_*H*_ = *p*_*mon*_*k*_*H*,*mon*_ + *p*_*dim*_*k*_*H*,*dim*_ + *p*_*trim*_*k*_*H*, *trim*_ given by the weighted sum of contributions from the monomeric form ([*η*]_*mon*_ and *k*_*H*,*mon*_), dimeric form ([*η*]_*dim*_ and *k*_*H*,*dim*_), and trimeric form ([*η*]_*trim*_ and *k*_*H*,*trim*_) with the monomer weight $p_{mon}=e^{-C/C_{\dim}}$ decreasing with *C*, dimer weight $p_{\dim}=\frac{C_{trim}}{C_{\dim}-C_{trim}}\left(e^{-C/C_{\dim}}-e^{-C/C_{trim}}\right)$ first increasing with *C* over the characteristic concentration for dimerization *C*_*dim*_ and then decreasing with *C* over some characteristic concentration for trimerization *C*_*trim*_, and the trimer weight *p*_*trim*_ = 1-*p*_*mon*_-*p*_*dim*_ increasing with *C* over the characteristic concentration for trimerization *C*_*trim*_.

## Data and code availability

All data are available from the corresponding authors upon reasonable request and are included in the main text and supplemental information.

## Acknowledgments

This work was conducted under a Sponsored Research Agreement between 10.13039/100014943Sarepta Therapeutics and 10.13039/100007919University of Massachusetts, Lowell.

## Author contributions

Y.C., formal analysis, methodology, visualization, investigation, writing ‒ original draft; I.N., formal analysis, methodology, visualization, investigation; D.P., formal analysis, investigation, visualization, writing ‒ original draft; K.A.M., conceptualization, formal analysis, investigation, methodology, supervision, validation, visualization, writing ‒ original draft; A.C., conceptualization, formal analysis, investigation, methodology, supervision, validation, visualization, writing ‒ original draft; V.B., conceptualization, formal analysis, investigation, methodology, supervision, validation, visualization, writing ‒ original draft.

## Declaration of interests

D.P. and A.C. are employees of Sarepta Therapeutics Inc. and own stock/options in the company.

## Supplemental information

Document S1. Figures S1–S15 and Tables S1–S3

Document S2. Article plus supplemental information
