# Supplementary material for: Kinetics, thermodynamics, and mechanisms of PMO interactions from computational molecular modeling
Source: Mol Ther Nucleic Acids. 2026 Aug 12;37(3):103056. doi: 10.1016/j.omtn.2026.103056 (PMC13524873; doi:10.1016/j.omtn.2026.103056)
Supplement: Supplementary file 1 [file mmc1.pdf]

OMTN, Volume *37*

Supplemental information

Kinetics, thermodynamics, and mechanisms of PMO interactions from computational molecular modeling

Ying Chou, Igor Novikov, Daniel Pierson, Kenneth A. Marx, Arani Chanda, and Valeri Barsegov

Supplemental Information

Supplemental Methods

Analysis of base-pairing, base-stacking and hydrogen bonds: The structure schematic for the calculation of the numbers of base pairs and base stackings is shown in Figure S1. First, we define the quantity, $\rho_{ij} = \sqrt{x_{kj}^2 + y_{kj}^2}$, and the x- and y-axes which are in the xy-plane of the base, whereas the z-axis is normal to the xy-plane. Here, x_{kj} and y_{kj} are the distances between the centers of mass of the two bases along the x- and y-axes, respectively. We also define z_{kj} to be the distance between the centers of mass of the two bases, and θ_{kj} to be the angle between the normal vectors of the two bases (Figure S1). A pair of bases is classified as stacked if $|z_{kj}|$ and $|z_{jk}| > 2$Å and ρ_{kj} or $\rho_{jk} < 2.5$Å and $|\theta_{kj}| < 40°$. All the non-stacked bases are considered to be base-paired if $|\theta_{kj}| < 60°$ and there exists at least one hydrogen bond between k-th and j-th bases (Figure S1)[1,2]. We assume that the hydrogen bond D–H…A between the hydrogen donor atom (D) and the hydrogen acceptor atom (A) is formed if the donor–acceptor distance d_{DA} is less than the 3.3Å cutoff distance and the bond angle (DHA) is larger than the 140° cutoff angle [1,2].

Supplemental Tables

Table S1. Molecular Mechanics parameters for covalent bonds and bond angles in PMOs: Shown for each covalent bond are the equilibrium covalent bond distance r_0 and spring constant k_b, and for each bond angle the equilibrium bond angle θ_0 and bending angle spring constant k_a. These force field parameters were determined for the PMOs comprising the oligonucleotide backbone explored in this study (see Material and Methods in the main text). The following atom types are described: CT – sp^3 hybridized carbon with 4 explicit substituents; NT – sp^3 hybridized nitrogen with 4 explicit substituents; H1 – hydrogen on aliphatic carbon with 1 electron-withdrawing group; OS – ester oxygen; O2 – phosphate oxygen; H – amide hydrogen; P – phosphorus in phosphate group; and HC – aliphatic carbon.

Bond	k_b, kcal/mol/Å	r_0, Å
CT-NT	326	1.465
CT-CT	310	1.526
CT-H1	340	1.090
CT-OS	320	1.410
P-O2	525	1.480
P-OS	230	1.610
NT-H	434	1.010
NT-P	367	1.670
Angle	k_θ, kcal/mol/rad²	θ_0, deg
NT-CT-CT	80.0	111.20
NT-CT-HC	49.5	109.88
CT-CT-HC	50.0	109.50
H1-CT-H1	35.0	109.50
CT-NT-CT	50.0	109.50
CT-NT-H	50.0	109.50
CT-CT-OS	50.0	109.50
OS-CT-H1	50.0	109.50
CT-OS-CT	60.0	109.50
P-OS-CT	100.0	120.50
CT-CT-CT	40.0	109.50
CT-NT-H	47.4	109.29
CT-NT-P	78.4	119.86
NT-P-O2	42.9	114.64
NT-P-OS	43.9	102.23
NT-P-NT	42.7	103.37

Table S2. Molecular Mechanics parameters for torsion angles in PMOs: Shown for each torsion angle are the number of bond paths and the magnitude of torsion energy $V_n/2$, the phase offset γ, and the periodicity of torsion n. Atom X denotes any atom of the atom type described in the caption to Table S1.

Torsion angle	no. of paths	$V_n/2$, kcal/mol	γ, deg	n
X–CT–CT–X	9	1.4	0.0	3.0
X–CT–NT–X	6	1.8	0.0	3.0
CT–NT–P–O2	1	3.0	180.0	-2.0
CT–OS–P–NT	3	2.4	0.0	2.0
CT–CT–NT–P	6	1.8	0.0	3.0
CT–NT–P–OS	6	18.0	180.0	2.0
CT–NT–P–NT	6	18.0	180.0	2.0
X–CM–P–X	6	8.8	180.0	2.0

Table S3. Dynamic molecular properties and thermodynamic characteristics for the 22-mer, 25-mer, and 30-mer PMO homodimers and heterodimers: Shown are the time-average quantities (and standard error of the mean) determined from the solution dimers for each PMO homodimers and heterodimers: the $RMSD$, PMO dimer size D, radius of gyration R_g , total number of intermolecular base pairs N_{BP}, total number of intermolecular base stacks N_{BS}, buried surface area $\Delta SASA$, and PMO-PMO interaction energy ΔE.

PMO		$RMSD$	D, nm	R_g, nm	N_{BP}	N_{BS}	$\Delta SASA$, Å^2	ΔE,kcal/mol
22-mer heterodimer	I-II	17.5±1.3	6.5±0.4	1.4±0.03	1.1±0.3	0.6±0.2	794±122	-33±6
	I-III	15.0±1.1	6.7±0.4	1.5±0.04	2.2±0.6	0.5±0.3	998±136	-49±8
	II-III	15.3±2	6.2±0.6	1.2±0.02	0.9±0.3	1.1±0.3	749±128	-32±6
22-mer homodimer	I-I	16.5±1.3	6.5±0.5	1.5±0.07	2.3±0.6	1±0.2	1,076±176	-50±9
	II-II	15.2±0.8	6.4±0.3	1.2±0.01	1±0.4	0.7±0.2	691±128	-32±7
	III-III	14.3±1.1	5.9±0.3	1.3±0.02	2±0.4	0.6±0.2	1,105±137	-50±6
25-mer heterodimer	I-II	15.7±0.8	6.3±0.2	1.8±0.04	1.8±0.5	0.8±0.2	826±104	-36±6
	I-III	16.6±0.7	6.3±0.2	1.8±0.1	0.8±0.2	0.8±0.2	1,048±146	-44±7
	II-III	16.6±1.1	6.3±0.2	1.8±0.03	1.4±0.2	1.6±0.4	1,209±108	-58±6
25-mer homodimer	I-I	15.1±1.2	6.3±0.2	1.8±0.1	1.1±0.4	0.6±0.2	840±175	-41±9
	II-II	16.0±1.2	5.8±0.2	1.7±0.04	1.9±0.3	1.3±0.2	1,225±118	-56±6
	III-III	16.4±0.9	7.0±0.3	1.9±0.1	1.5±0.4	1.0±0.4	1,014±133	-49±7
30-mer heterodimer	I-II	18.7±1.1	6.6±0.3	1.9±0.1	1.4±0.3	0.5±0.2	991±132	-39±5
	I-III	21.2±1.6	6.3±0.2	1.8±0.04	2.3±0.5	1.1±0.3	1,611±157	-67±8
	II-III	16.7±1.2	6.6±0.2	1.9±0.04	2.0±0.3	0.9±0.3	1,117±123	-46±6
30-mer homodimer	I-I	16.7±1.2	6.6±0.2	1.9±0.1	1.6±0.4	0.7±0.2	1,012±106	-43±5
	II-II	18.1±1.0	6.9±0.3	1.9±0.1	3.0±0.6	0.4±0.1	849±121	-43±6
	III-III	20.2±1.3	7.3±0.3	2.0±0.1	2.1±0.5	1.3±0.3	1,229±123	-53±6

Supplemental Figures

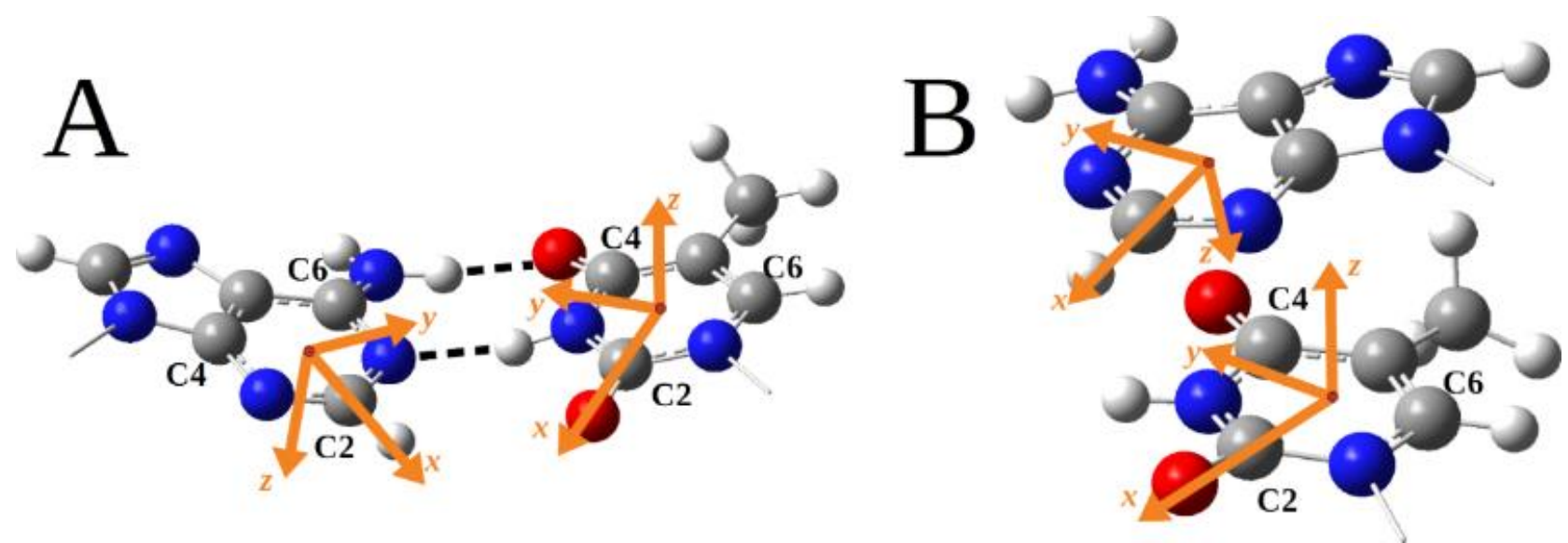

Figure S1. Base pairing and base stacking interactions in PMO molecules: Panel **A:** local coordinate systems for purines and pyrimidines used in the calculation of base pairing and base stacking interactions (as described in Supplemental Methods). In the center of the base ring, with C2-, C4-, and C6-atoms represents the origin of the coordinate system (as displayed in the graph). The x-and y-axes lie in the plane of the base whereas the z-axis is normal to the xy-plane. The x-axis is pointed in the direction of C2-atom, and the y-axis is pointed toward the C6-atom (for A and G) and toward the C4-atom (for C and U). Two bases are forming a base pair via hydrogen bonds shown as dashed black lines. Panel **B**: The same x-, y- and z-axes displayed in panel **A** were used to describe formation of base stacking interactions.

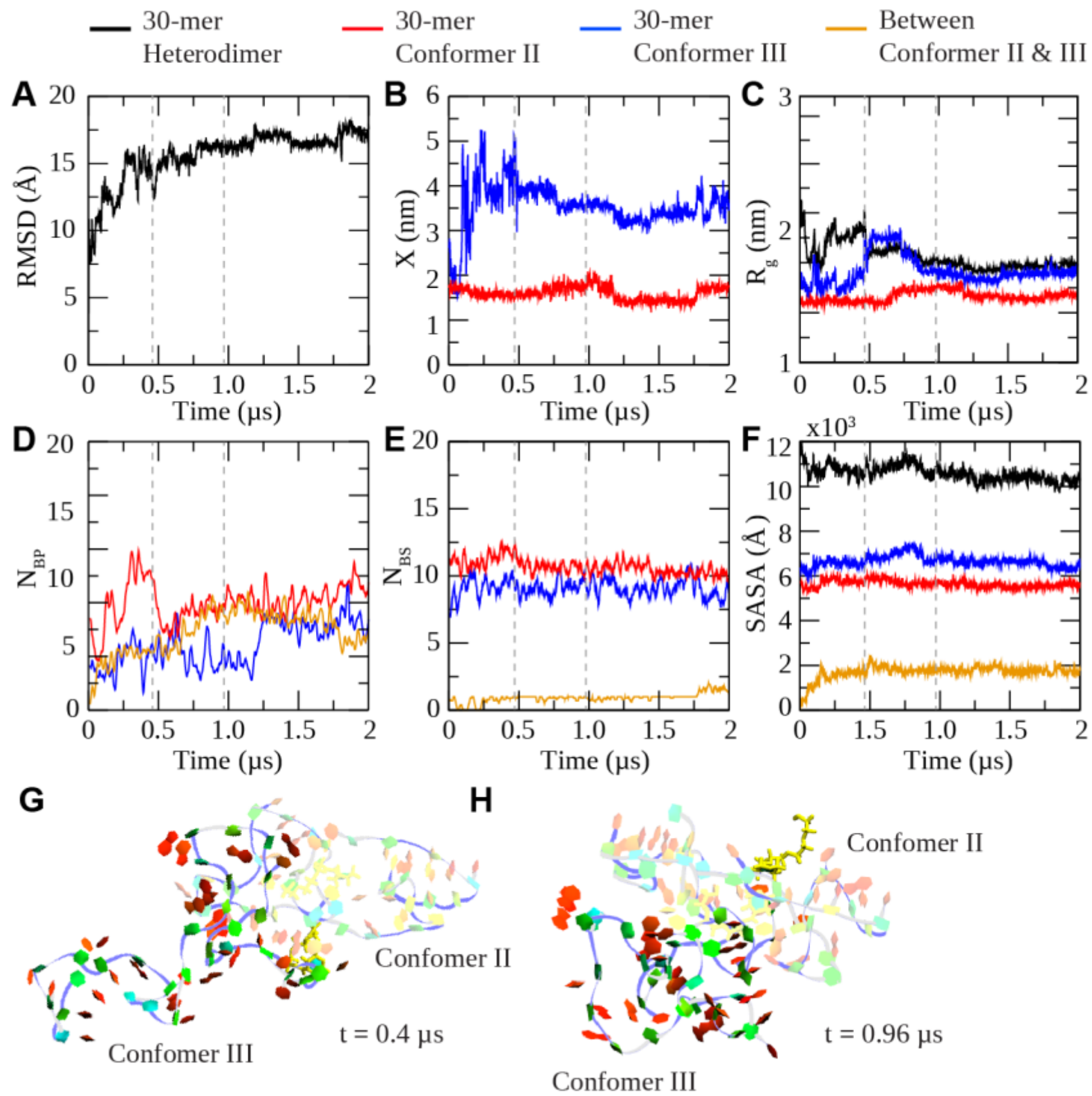

Figure S2. Dynamic molecular properties and structure snapshots for representative 30-mer heterodimer system (conformers II and III): Displayed are the time profiles of the root-mean-square deviation (RMSD) (panel **A**), end-to-end distance X (panel **B**), radius of gyration R_g (panel **C**), number of base pairs N_{BP} (panel **D**), number of base stacking interactions N_{BS} (panel **E**), and solvent-accessible surface area ($SASA$) (panel **F**). Color denotations for molecular properties associated with the dimer, conformers II and III, and interactions between conformers II and III are explained on the graph. Also shown are structure snapshots showing interactions between conformer II (pastel color) and conformer III (non-pastel color) in paper chain and twister representation at 0.4 μs (panel **G**), and at 0.96 μs time (panel **H**). Dotted grey lines in panels **A-F** indicate the times at which the snapshots were taken. The PMO backbones are shown in Twister representation (blue), and nucleobases as paper chains (red and green); TEG piperazine end cap shown as yellow licorice.

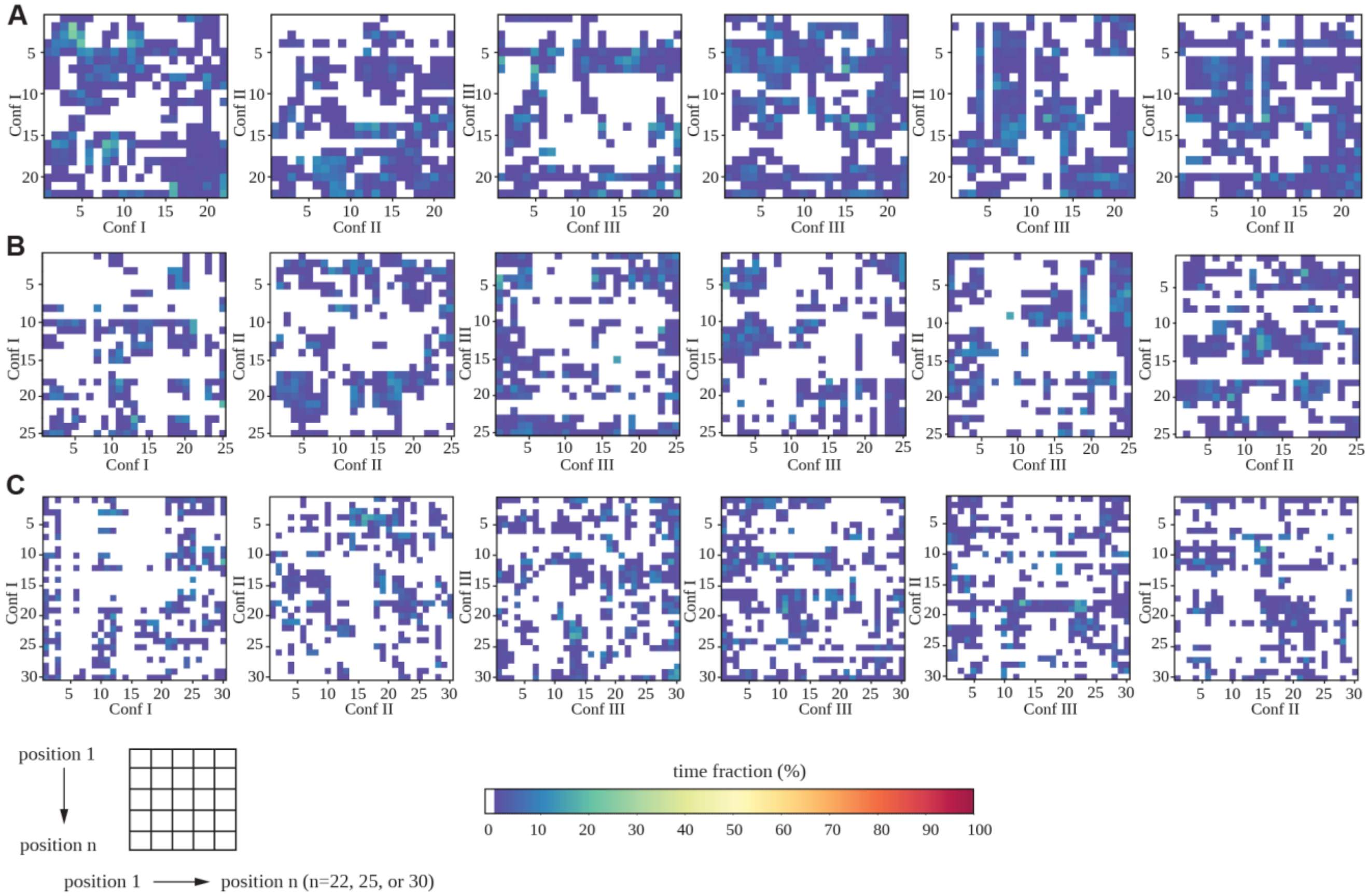

Figure S3. Persistence maps of intermolecular base-base contacts using 10Å distance threshold in PMO-PMO dimers for the 25-mer and 30-mer sequences. Contact maps are shown for dimers involving the three most representative conformers I, II, and III of 22-mer (panel **A**), 25-mer (panel **B**), and 30-mer (panel **C**) PMOs. Each pixel in each matrix represents a pairwise contact between nucleobases from opposing monomers, with the color scale indicating the average contact persistence. Persistence is defined as a fraction of total simulation time during which a given contact was present. Contacts that never formed are shown in white. PMO bases are numbered from the 5′ end. These maps illustrate the transient and heterogeneous nature of intermolecular interactions, with the majority of contacts persisting for less than 10% of the simulation time (i.e. 0.2 µs).

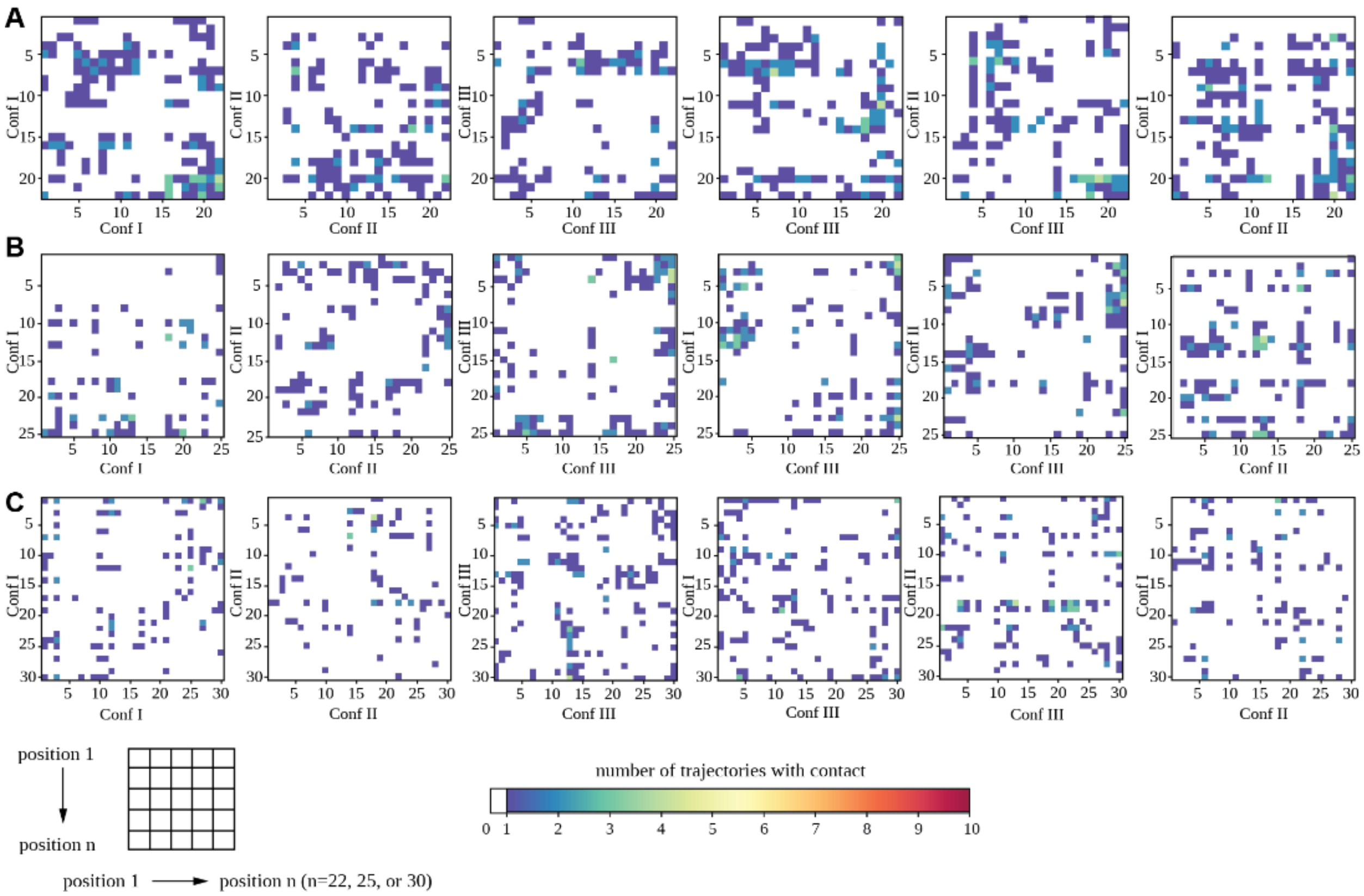

Figure S4. Intermolecular interaction maps (using 5Å distance threshold) of PMO-PMO dimers for 22-mer, 25-mer, and 30-mer sequences. Contact maps are shown between morpholino nucleobases for the three most representative conformers (I, II, and III) of 22-mer (panel **A**), 25-mer (panel **B**), and 30-mer PMOs (panel **C**). Each map displays nucleobase-nucleobase contacts as pixels in a matrix format. The color scale indicates how many of the 10 independent simulation trajectories of a given PMO dimer exhibited a contact formation between each pair of positions (a maximum value of 10 indicates that the nucleobase pair in question was in contact in all 10 trajectories). A contact is counted if the pair of nucleobases were within the 5Å distance at any point during a given trajectory. PMO bases are numbered from the 5′ end of the sequence.

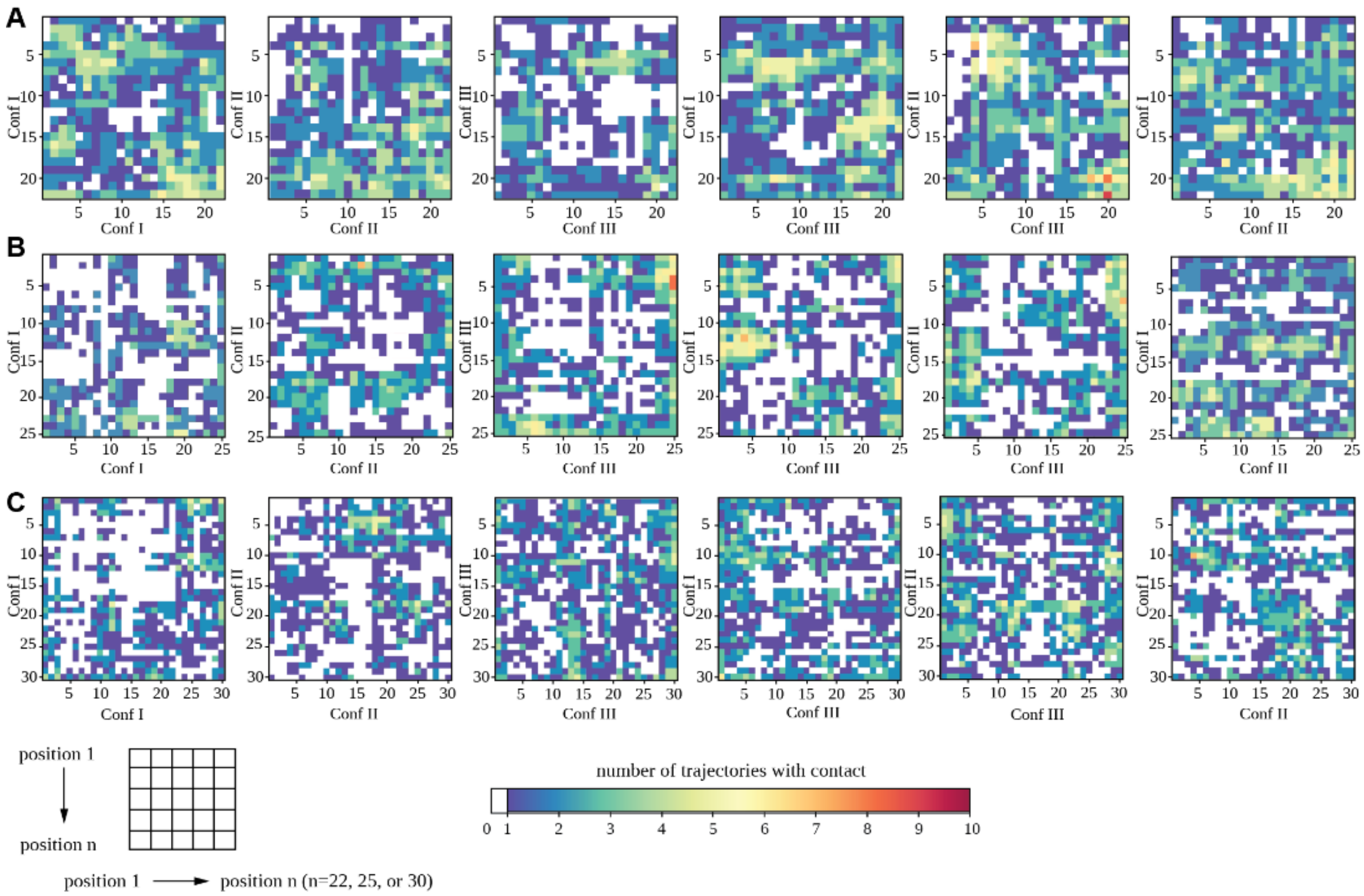

Figure S5. Intermolecular interaction maps (using 10Å distance threshold) of PMO-PMO dimers for 22-mer, 25-mer, and 30-mer sequences. Contact maps are shown between morpholino nucleobases for the three most representative conformers (I, II, and III) of 22-mer (panel **A**), 25-mer (panel **B**), and 30-mer PMOs (panel **C**). Each map displays nucleobase-nucleobase contacts as pixels in a matrix format. The color scale indicates how many of the 10 independent simulation trajectories of a given PMO dimer exhibited a contact formation between each pair of positions (a maximum value of 10 indicates that the nucleobase pair in question was in contact in all 10 trajectories). A contact is counted if the pair of nucleobases were within the 10Å distance at any point during a given trajectory. PMO bases are numbered from the 5′ end of the sequence.

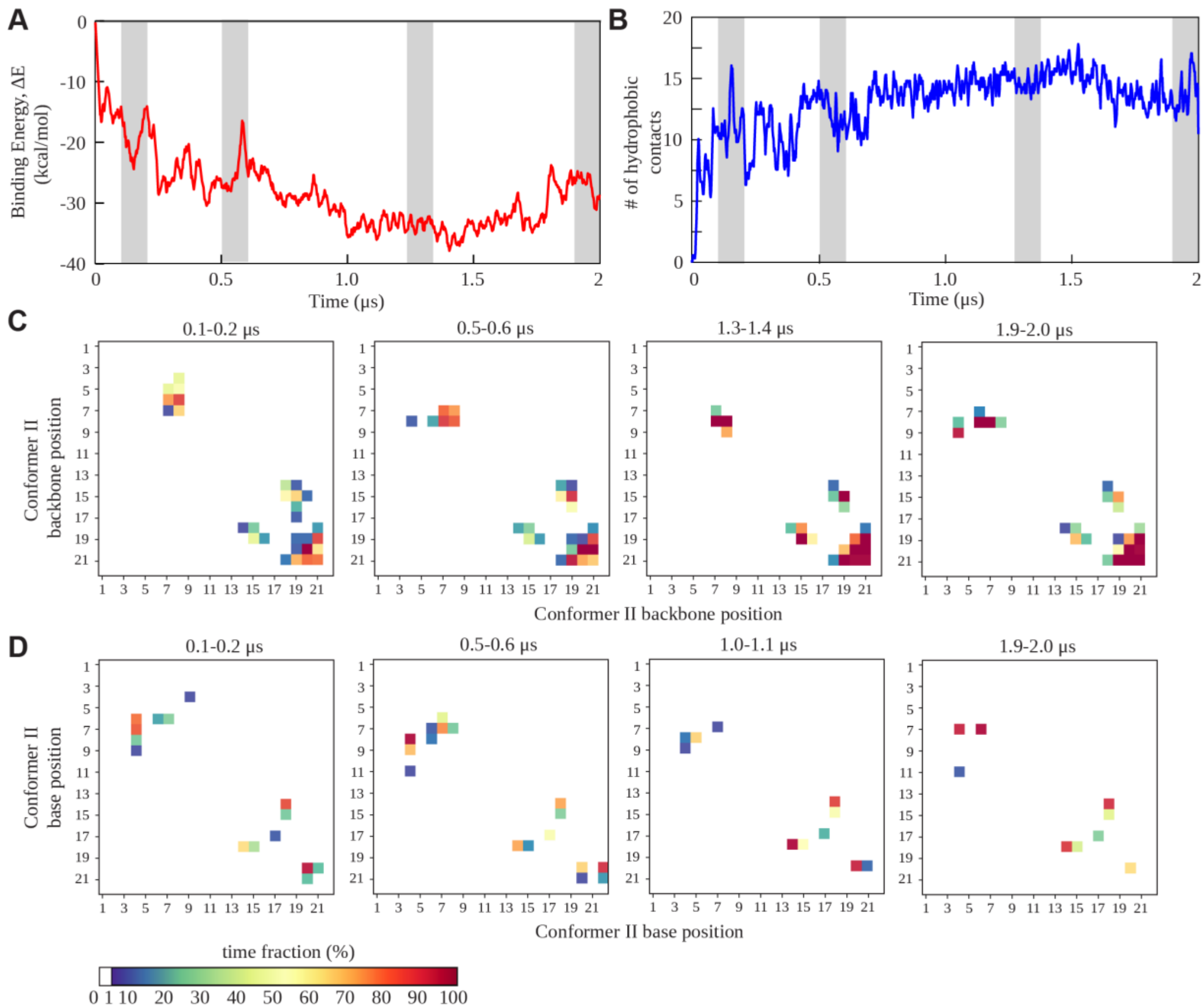

Figure S6. Interaction energies and backbone-mediated hydrophobic contact persistence (using 7.5Å distance threshold) for a representative trajectory of the 22-mer homodimer II-II. Shown are the time-dependent interaction energy between the two monomers over the course of the trajectory (panel **A**), the number of hydrophobic contacts during the trajectory (panel **B**), and the persistence of hydrophobic backbone contacts calculated within 100-ns time windows (panel **C**). Hydrophobic contacts are defined as interactions within a 7.5Å cutoff between phosphorodiamidate and morpholino groups, including all pairwise combinations: phosphorodiamidate–morpholino, morpholino–morpholino, phosphorodiamidate–phosphorodiamidate, and morpholino–phosphorodiamidate. Also shown is the persistence of base–base contacts within the same 100-ns intervals (panel **D**), defined as interactions between nucleobases of the two monomers within a 7.5Å cutoff between their C5-atoms.

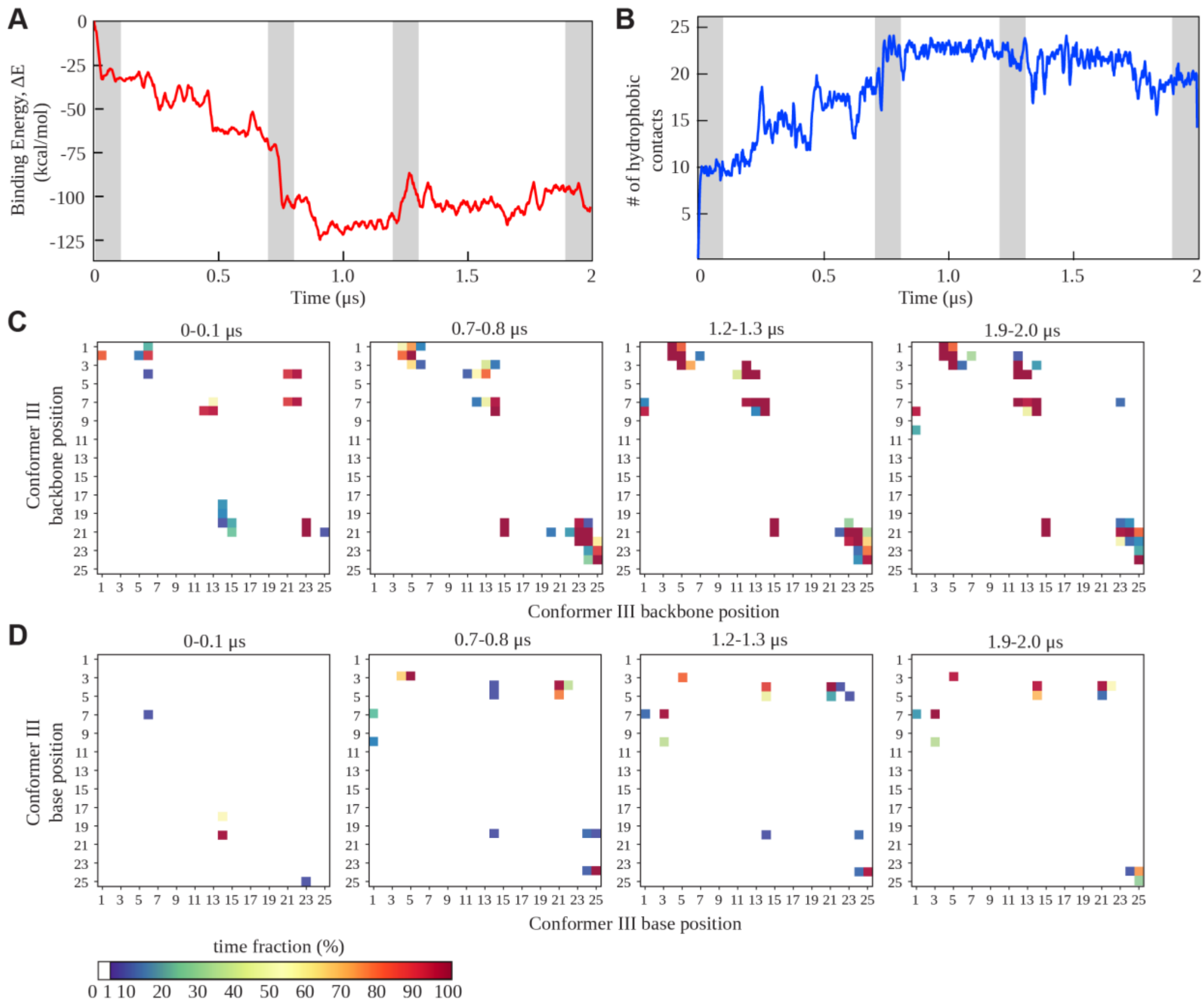

Figure S7. Interaction energies and backbone-mediated hydrophobic contact persistence (using 7.5Å distance threshold) for a representative trajectory of the 25-mer homodimer III–III. Shown are the time-dependent interaction energy between the two monomers over the course of the trajectory (panel **A**), the number of hydrophobic contacts during the trajectory (panel **B**), and the persistence of hydrophobic backbone contacts calculated within 100-ns time windows (panel **C**). Hydrophobic contacts are defined as interactions within a 7.5Å cutoff between phosphorodiamidate and morpholino groups, including all pairwise combinations: phosphorodiamidate–morpholino, morpholino–morpholino, phosphorodiamidate–phosphorodiamidate, and morpholino–phosphorodiamidate. Also shown is the persistence of base–base contacts within the same 100-ns intervals (panel **D**), defined as interactions between nucleobases of the two monomers within a 7.5Å cutoff between their C5-atoms.

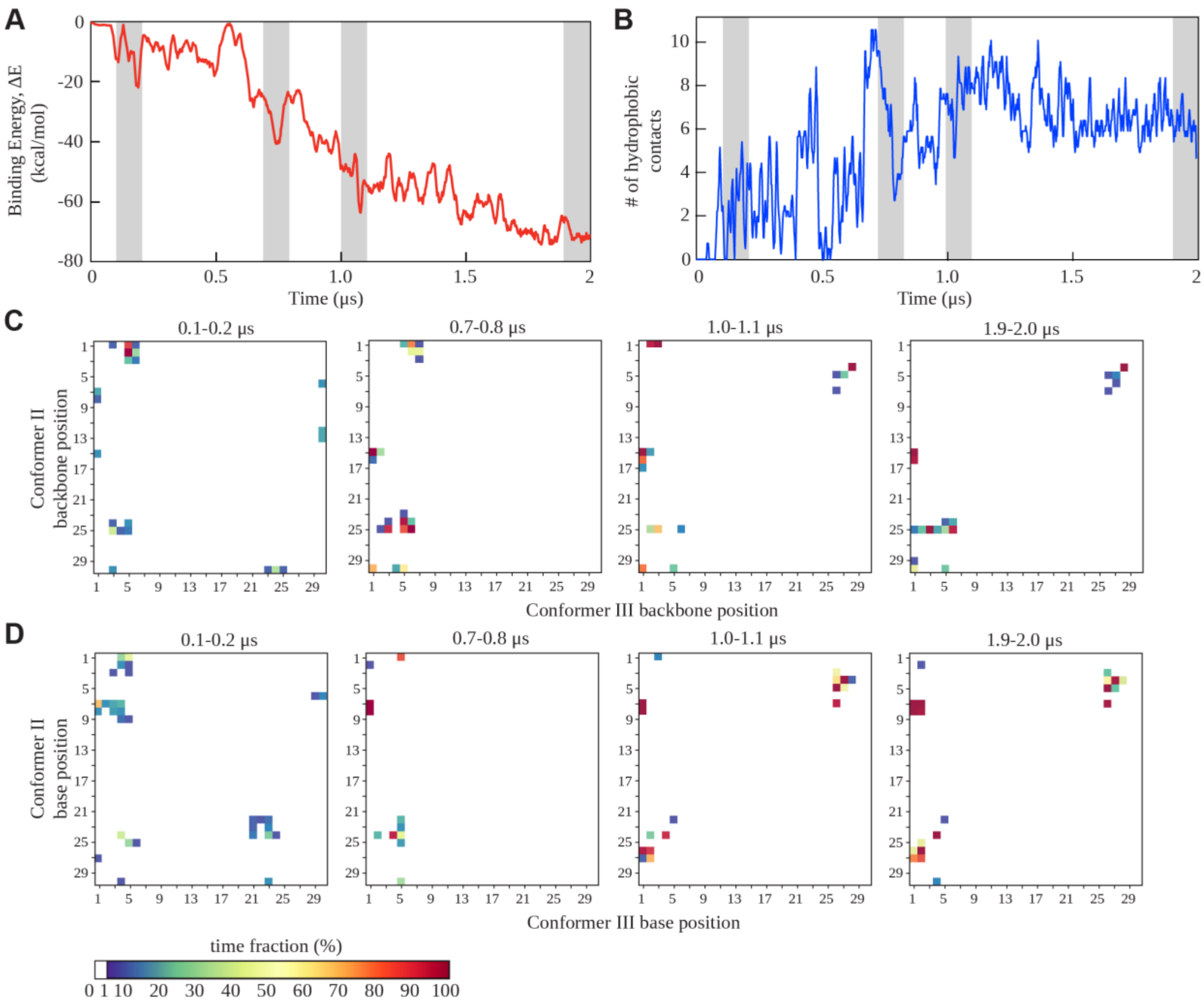

Figure S8. Interaction energies and backbone-mediated hydrophobic contact persistence (using 7.5Å distance threshold) for a representative trajectory of the 30-mer heterodimer II-III. Shown are the time-dependent interaction energy between the two monomers over the course of the trajectory (panel **A**), the number of hydrophobic contacts during the trajectory (panel **B**), and the persistence of hydrophobic backbone contacts calculated within 100-ns time windows (panel **C**). Hydrophobic contacts are defined as interactions within a 7.5Å cutoff between phosphorodiamidate and morpholino groups, including all pairwise combinations: phosphorodiamidate–morpholino, morpholino–morpholino, phosphorodiamidate–phosphorodiamidate, and morpholino–phosphorodiamidate. Also shown is the persistence of base–base contacts within the same 100-ns intervals (panel **D**), defined as interactions between nucleobases of the two monomers within a 7.5Å cutoff between their C5-atoms.

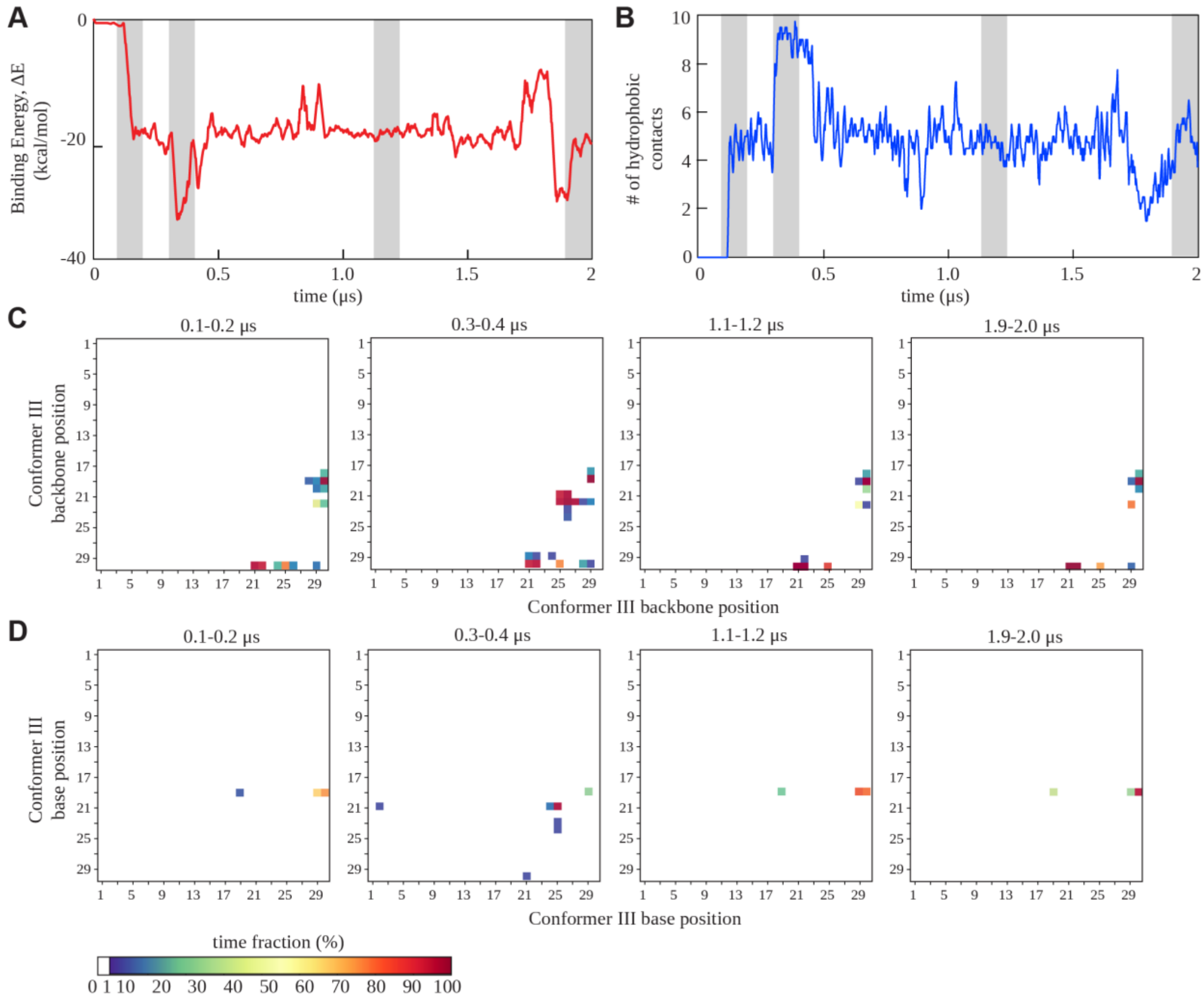

Figure S9. Interaction energies and backbone-mediated hydrophobic contact persistence (using 7.5Å distance threshold) for a representative trajectory of the 30-mer homodimer III–III. Shown are the time-dependent interaction energy between the two monomers over the course of the trajectory (panel **A**), the number of hydrophobic contacts during the trajectory (panel **B**), and the persistence of hydrophobic backbone contacts calculated within 100-ns time windows (panel **C**). Hydrophobic contacts are defined as interactions within a 7.5Å cutoff between phosphorodiamidate and morpholino groups, including all pairwise combinations: phosphorodiamidate–morpholino, morpholino–morpholino, phosphorodiamidate–phosphorodiamidate, and morpholino–phosphorodiamidate. Also shown is the persistence of base–base contacts within the same 100-ns intervals (panel **D**), defined as interactions between nucleobases of the two monomers within a 7.5 Å cutoff between their C5-atoms.

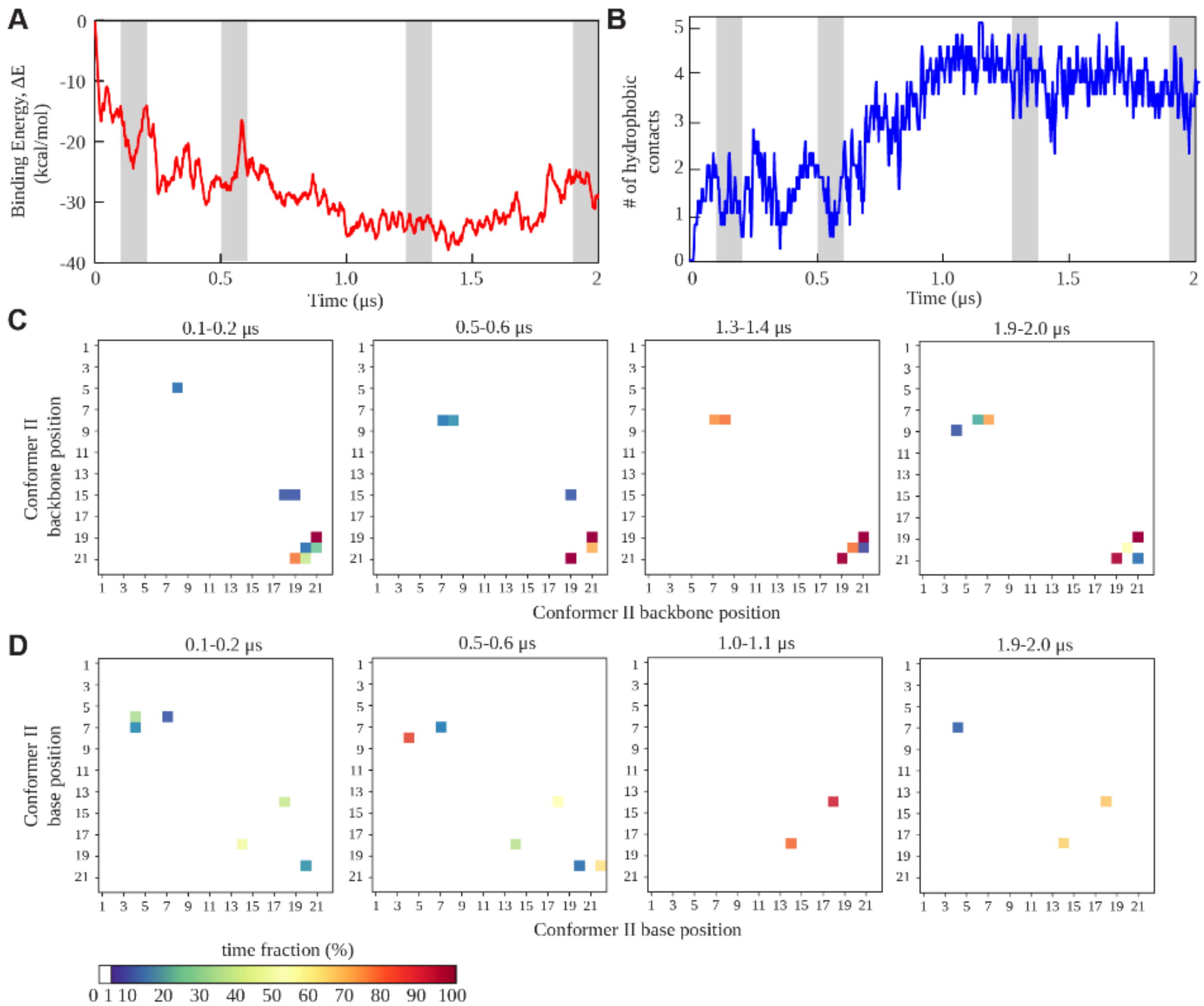

Figure S10. Interaction energies and backbone-mediated hydrophobic contact persistence (using 5Å distance threshold) for a representative trajectory of the 22-mer homodimer II-II. Shown are the time-dependent interaction energy between the two monomers over the course of the trajectory (panel **A**), the number of hydrophobic contacts observed (panel **B**), and the persistence of hydrophobic backbone contacts in 100-ns time windows (panel **C**). Hydrophobic contacts are defined as interactions within the 5Å cutoff between phosphorodiamidate and morpholino groups, including all pairwise combinations: phosphorodiamidate–morpholino, morpholino–morpholino, phosphorodiamidate–phosphorodiamidate, and morpholino–phosphorodiamidate. Panel **D** shows the persistence of base–base contacts within the same 100-ns intervals, defined as interactions between nucleobases of the two monomers within the 5Å cutoff between their C5-atoms.

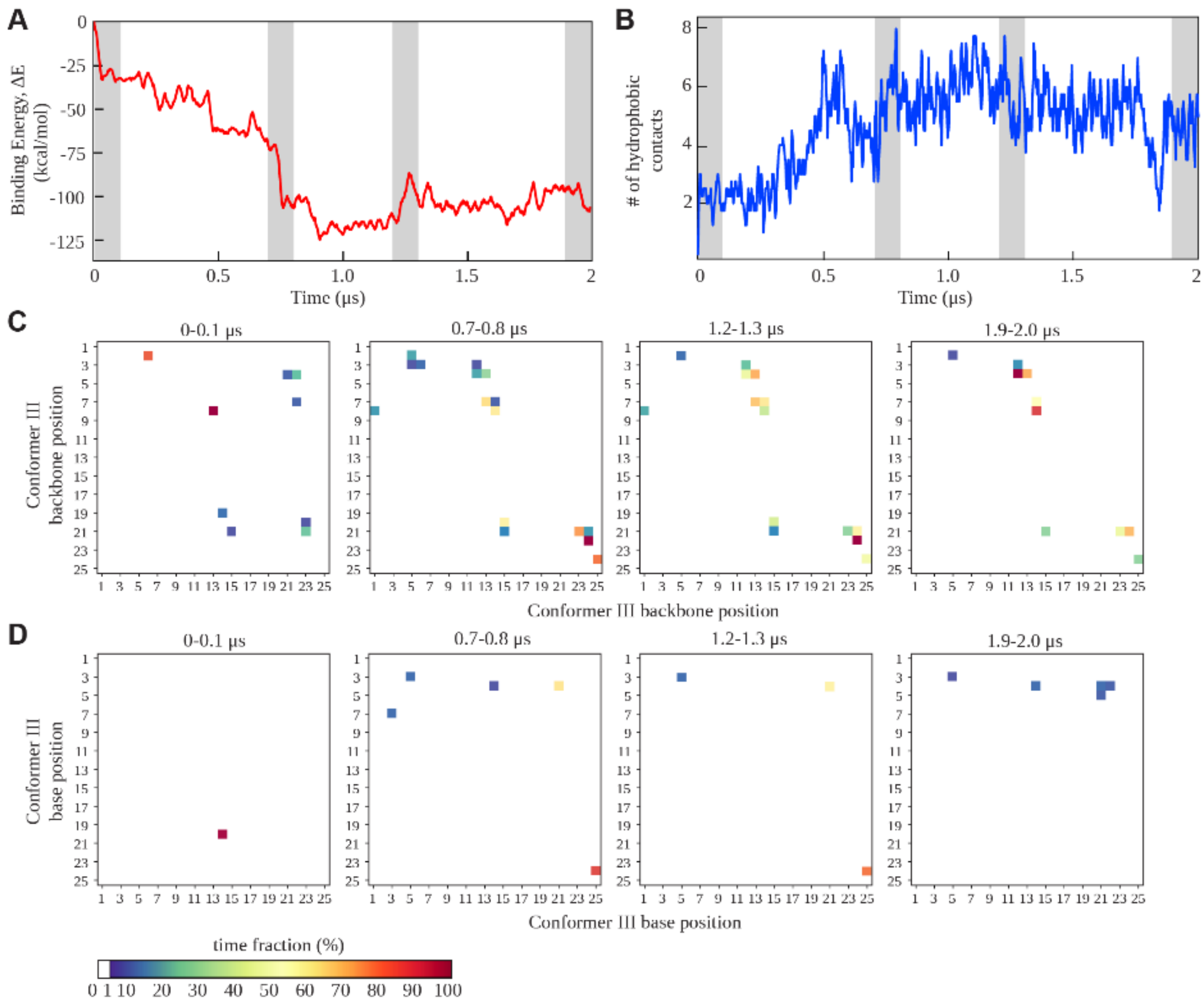

Figure S11. Interaction energies and backbone-mediated hydrophobic contact persistence (using 5Å distance threshold) for a representative trajectory of the 25-mer homodimer III–III. Shown are the time-dependent interaction energy between the two monomers over the course of the trajectory (panel **A**), the number of hydrophobic contacts observed (panel **B**), and the persistence of hydrophobic backbone contacts in 100-ns time windows (panel **C**). Hydrophobic contacts are defined as interactions within the 5Å cutoff between phosphorodiamidate and morpholino groups, including all pairwise combinations: phosphorodiamidate–morpholino, morpholino–morpholino, phosphorodiamidate–phosphorodiamidate, and morpholino–phosphorodiamidate. Panel **D** shows the persistence of base–base contacts within the same 100-ns intervals, defined as interactions between nucleobases of the two monomers within the 5Å cutoff between their C5-atoms.

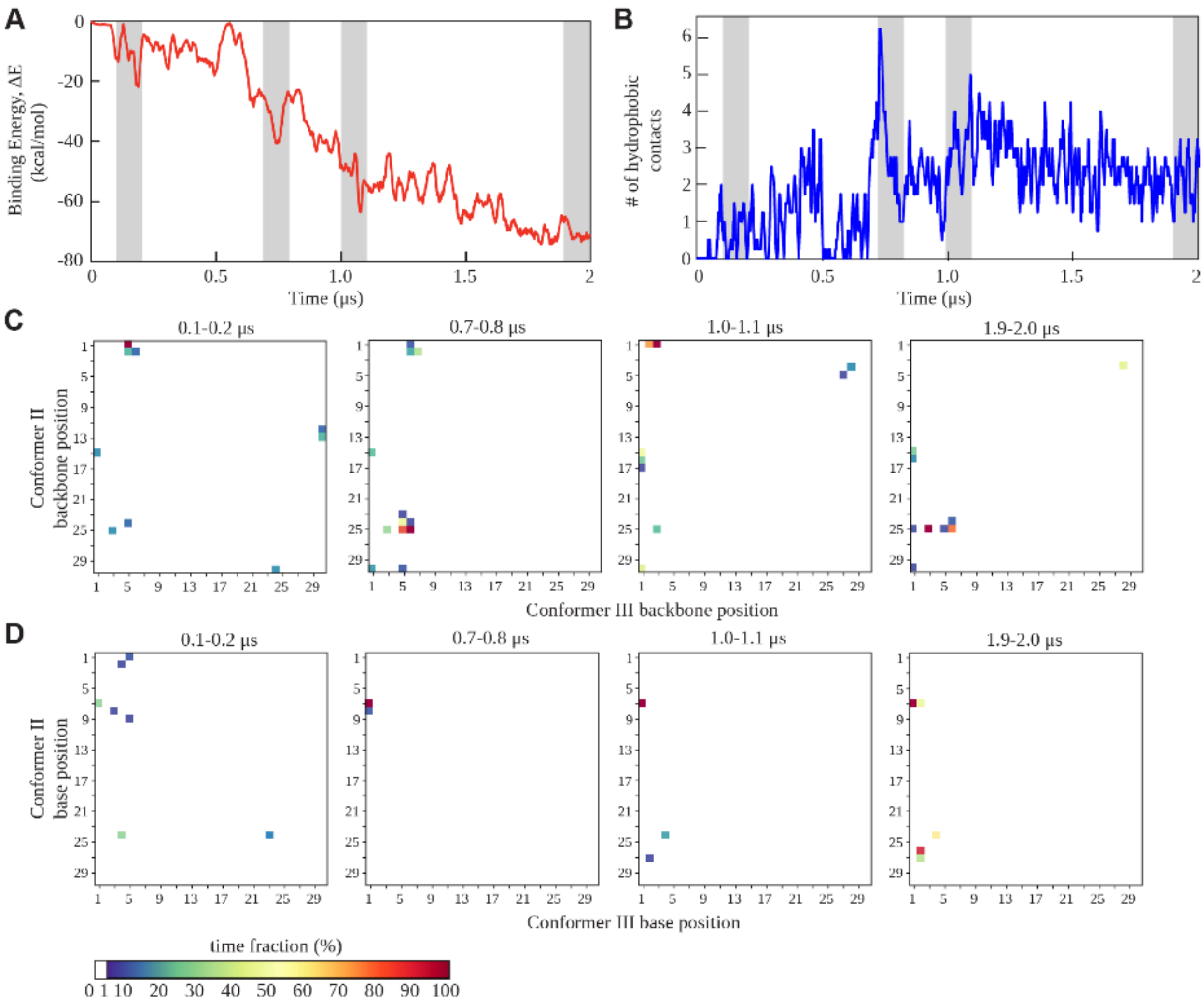

Figure S12. Interaction energies and backbone-mediated hydrophobic contact persistence (using 5Å distance threshold) for a representative trajectory of the 30-mer heterodimer II–III. Shown are the time-dependent interaction energy between the two monomers over the course of the trajectory (panel **A**), the number of hydrophobic contacts observed (panel **B**), and the persistence of hydrophobic backbone contacts in 100-ns time windows (panel **C**). Hydrophobic contacts are defined as interactions within the 5Å cutoff between phosphorodiamidate and morpholino groups, including all pairwise combinations: phosphorodiamidate–morpholino, morpholino–morpholino, phosphorodiamidate–phosphorodiamidate, and morpholino–phosphorodiamidate. Panel **D** shows the persistence of base–base contacts within the same 100-ns intervals, defined as interactions between nucleobases of the two monomers within the 5Å cutoff between their C5-atoms.

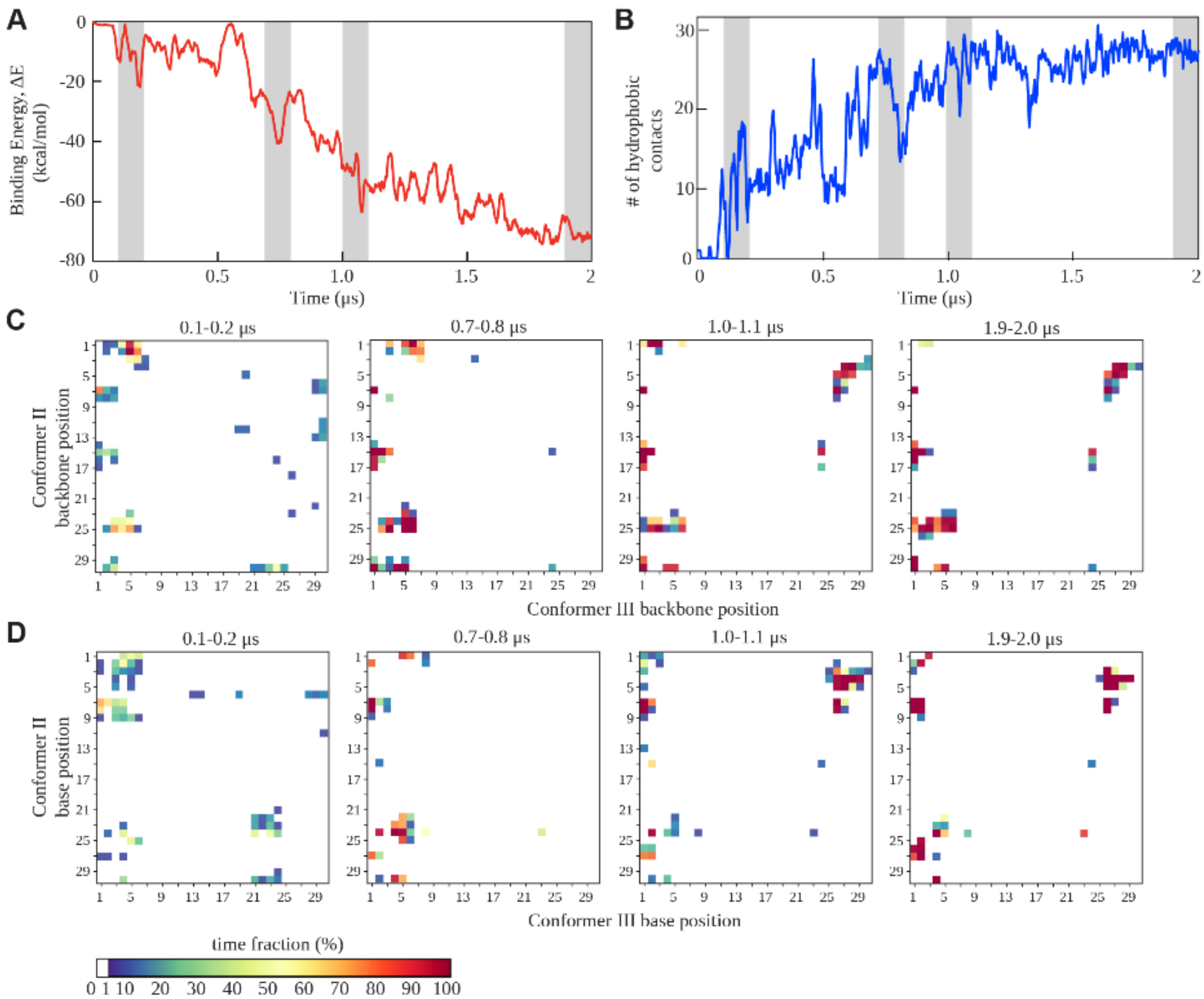

Figure S13. Interaction energies and backbone-mediated hydrophobic contact persistence (using 10Å distance threshold) for a representative trajectory of the 22-mer homodimer II–II. Shown are the time-dependent interaction energy between the two monomers over the course of the trajectory (panel **A**), the number of hydrophobic contacts observed (panel **B**), and the persistence of hydrophobic backbone contacts in 100-ns time windows (panel **C**). Hydrophobic contacts are defined as interactions within the 10Å cutoff between phosphorodiamidate and morpholino groups, including all pairwise combinations: phosphorodiamidate–morpholino, morpholino–morpholino, phosphorodiamidate–phosphorodiamidate, and morpholino–phosphorodiamidate. Panel **D** shows the persistence of base–base contacts within the same 100-ns intervals, defined as interactions between nucleobases of the two monomers within the 10Å cutoff between their C5-atoms.

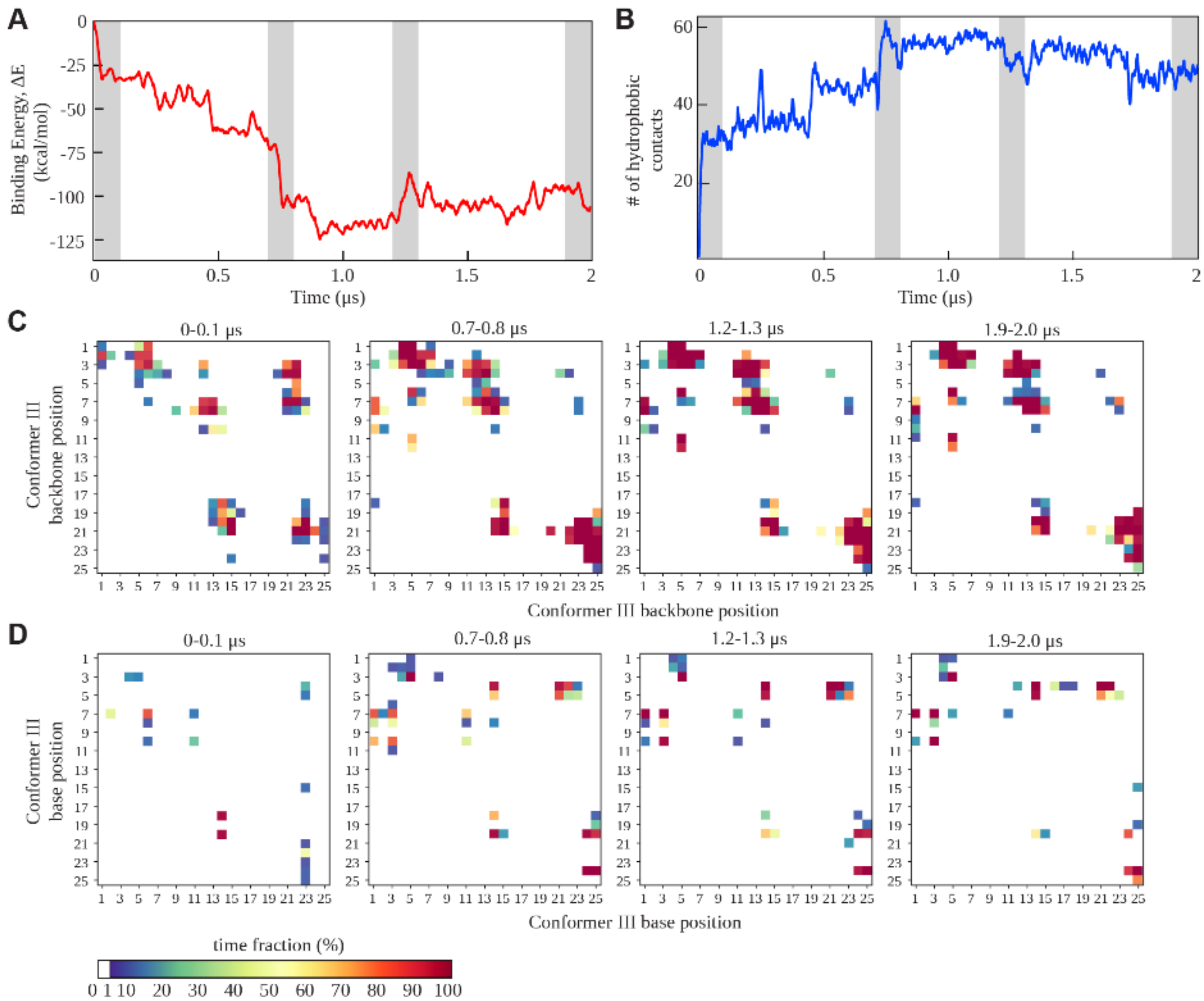

Figure S14. Interaction energies and backbone-mediated hydrophobic contact persistence (using 10Å distance threshold) for a representative trajectory of the 25-mer homodimer III–III. Shown are the time-dependent interaction energy between the two monomers over the course of the trajectory (panel **A**), the number of hydrophobic contacts observed (panel **B**), and the persistence of hydrophobic backbone contacts in 100-ns time windows (panel **C**). Hydrophobic contacts are defined as interactions within the 10Å cutoff between phosphorodiamidate and morpholino groups, including all pairwise combinations: phosphorodiamidate–morpholino, morpholino–morpholino, phosphorodiamidate–phosphorodiamidate, and morpholino–phosphorodiamidate. Panel **D** shows the persistence of base–base contacts within the same 100-ns intervals, defined as interactions between nucleobases of the two monomers within the 10Å cutoff between their C5-atoms.

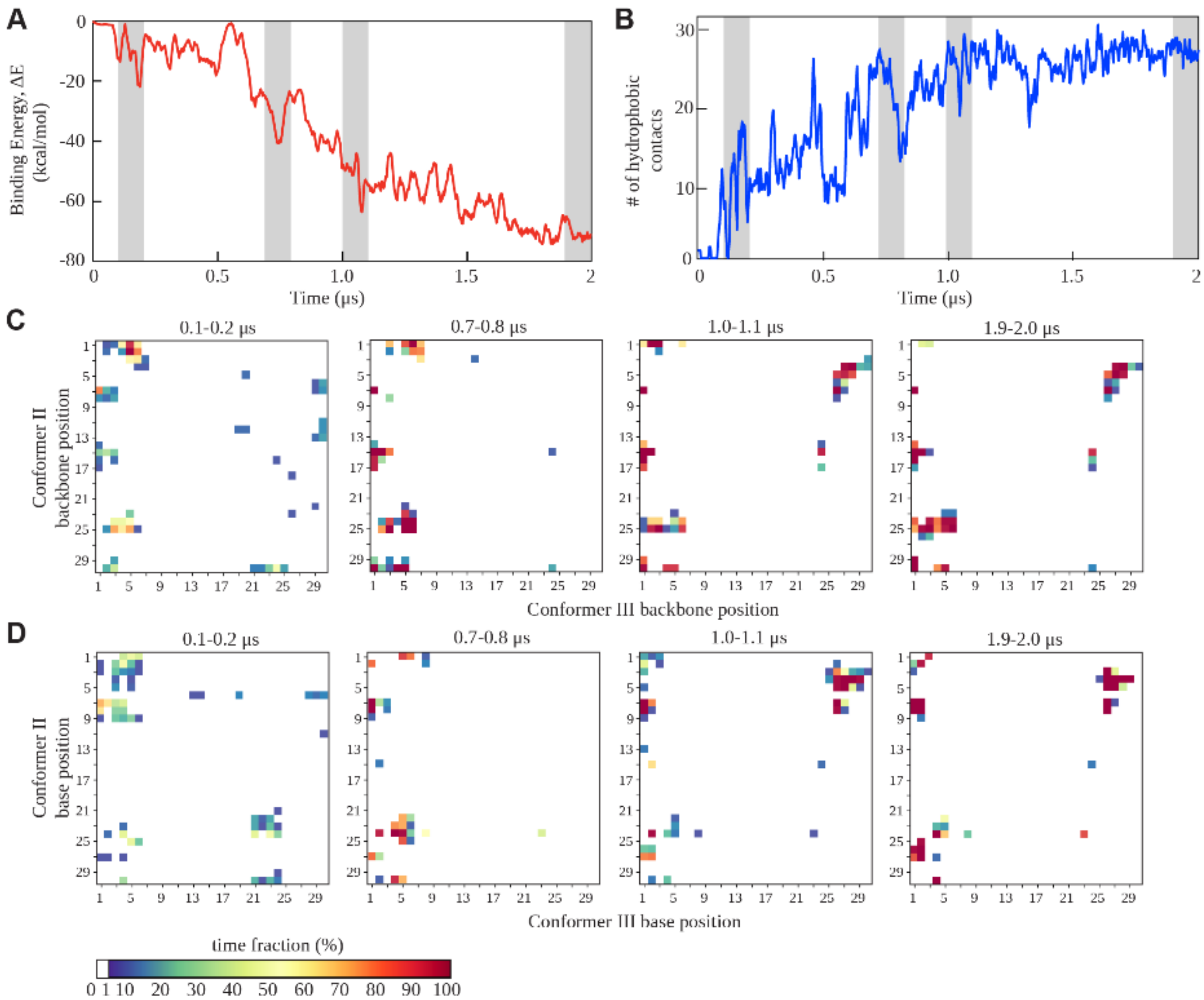

Figure S15. Interaction energies and backbone-mediated hydrophobic contact persistence (using 10Å distance threshold) for a representative trajectory of the 30-mer heterodimer II–III. Shown are the time-dependent interaction energy between the two monomers over the course of the trajectory (panel **A**), the number of hydrophobic contacts observed (panel **B**), and the persistence of hydrophobic backbone contacts in 100-ns time windows (panel **C**). Hydrophobic contacts are defined as interactions within the 10Å cutoff between phosphorodiamidate and morpholino groups, including all pairwise combinations: phosphorodiamidate–morpholino, morpholino–morpholino, phosphorodiamidate–phosphorodiamidate, and morpholino–phosphorodiamidate. Panel **D** shows the persistence of base–base contacts within the same 100-ns intervals, defined as interactions between nucleobases of the two monomers within the 10Å cutoff between their C5-atoms.

Supplemental Movies

Movie S1. Interaction between 22-mer PMO conformers I and III during a 2-μs MD simulation at 300 K in explicit water (ice-blue transparent spheres). PMO backbones are shown in Twister representation (blue), and nucleobases as paper chains (red and green). Conformer I is shown with pastel blue backbone to distinguish it from conformer III (regular blue). The movie is 65 seconds long and played 4.4×10^7 times slower than computational experiment. We observe repeated association, dissociation, and re-association events between PMO molecules.

Movie S2. Interaction between 25-mer PMO conformers I and II during a 2-μs MD simulation at 300 K in explicit water (ice-blue transparent spheres). PMO backbones are shown in Twister representation (blue), nucleobases as paper chains (red and green), and 5'-end TEG cap as licorice (yellow). Conformer I is shown with pastel blue backbone to distinguish it from conformer II (blue backbone). The movie is 33 seconds long and played 2.2×10^7 times slower than computational experiment. We observe structural rearrangement, suggesting transient and flexible interactions between PMO conformers.

Movie S3. Interaction between 30-mer PMO conformers II and III during a 2-μs MD simulation at 300 K in explicit water (ice-blue transparent spheres). PMO backbones are shown in Twister representation (blue), nucleobases as paper chains (red and green), and 5'-end TEG cap as licorice (yellow). Conformer II is shown with pastel blue backbone to distinguish it from conformer III (blue backbone). The movie is 25 seconds long and played 1.6×10^7 time slower than computational experiment. We observed unfolding and refolding of Conformer II.

Supplemental References:

1. Maksudov, F., Kliuchnikov, E., Pierson, D., Ujwal, M.L., Marx, K.A., Chanda, A., and Barsegov, V. (2023). Therapeutic phosphorodiamidate morpholino oligonucleotides: Physical properties, solution structures, and folding thermodynamics. Mol Ther Nucleic Acids *31*, 631–647. https://doi.org/10.1016/j.omtn.2023.02.007.
2. Kliuchnikov, E., Maksudov, F., Pierson, D., Marx, K.A., Chanda, A., and Barsegov, V. (2025). Molecular properties and intramolecular interactions of peptide-conjugated phosphorodiamidate morpholino oligonucleotides. Mol. Ther. Nucleic Acids *36*, 102685. https://doi.org/https://doi.org/10.1016/j.omtn.2025.102685.
